# Engineering transcriptional regulation of pentose metabolism in *Rhodosporidium* *toruloides* for improved conversion of xylose to bioproducts

**DOI:** 10.1186/s12934-023-02148-5

**Published:** 2023-08-03

**Authors:** Samuel T. Coradetti, Paul A. Adamczyk, Di Liu, Yuqian Gao, Peter B. Otoupal, Gina M. Geiselman, Bobbie-Jo M. Webb-Robertson, Meagan C. Burnet, Young-Mo Kim, Kristin E. Burnum-Johnson, Jon Magnuson, John M. Gladden

**Affiliations:** 1 DOE Agile Biofoundry, 5885 Hollis Street, Fourth Floor, Emeryville, CA 94608 USA; 2 Sandia National Laboratories, Livermore, CA USA; 3 Present Address: Agricultural Research Service, United States Department of Agriculture, Ithaca, NY USA; 4 Pacific Northwest National Laboratory, Richland, WA USA; 5 Joint BioEnergy Institute, Emeryville, CA USA

**Keywords:** *Rhodosporidiumtoruloides*, Fatty alcohol, Xylose metabolism, Carbon catabolite repression, Transcriptional regulation, Proteomics

## Abstract

Efficient conversion of pentose sugars remains a significant barrier to the replacement of petroleum-derived chemicals with plant biomass-derived bioproducts. While the oleaginous yeast *Rhodosporidium* *toruloides* (also known as *Rhodotorula* *toruloides*) has a relatively robust native metabolism of pentose sugars compared to other wild yeasts, faster assimilation of those sugars will be required for industrial utilization of pentoses. To increase the rate of pentose assimilation in *R.* *toruloides,* we leveraged previously reported high-throughput fitness data to identify potential regulators of pentose catabolism. Two genes were selected for further investigation, a putative transcription factor (RTO4_12978, Pnt1) and a homolog of a glucose transceptor involved in carbon catabolite repression (RTO4_11990). Overexpression of Pnt1 increased the specific growth rate approximately twofold early in cultures on xylose and increased the maximum specific growth by 18% while decreasing accumulation of arabitol and xylitol in fast-growing cultures. Improved growth dynamics on xylose translated to a 120% increase in the overall rate of xylose conversion to fatty alcohols in batch culture. Proteomic analysis confirmed that Pnt1 is a major regulator of pentose catabolism in *R.* *toruloides.* Deletion of RTO4_11990 increased the growth rate on xylose, but did not relieve carbon catabolite repression in the presence of glucose. Carbon catabolite repression signaling networks remain poorly characterized in *R.* *toruloides* and likely comprise a different set of proteins than those mainly characterized in ascomycete fungi.

**Supplementary Information:**

The online version contains supplementary material available at 10.1186/s12934-023-02148-5.

## Introduction

Pentose sugars, primarily xylose and arabinose, are a significant fraction of lignocellulosic biomass [1]. Any sustainable and economically competitive implementation of an integrated biorefinery to displace petroleum-derived chemicals will require efficient utilization of those sugars, yet efficient bioconversion of pentoses to value-added chemicals remains a major unsolved challenge [2, 3].

The oleaginous yeast *Rhodosporidium* *toruloides* (also known as *Rhodotorula* *toruloides*) consumes a wide range of carbon compounds derived from plant biomass, including six carbon sugars, five carbon sugars, and lignin breakdown products [4]. *R.* *toruloides* is also relatively tolerant of many biomass breakdown products that inhibit the growth of other microorganisms [5] and even more robust strains have been isolated through adaptive laboratory evolution [6, 7]. This extensive catabolic capability plus its native biosynthetic capacity for lipids and carotenoids make it an attractive platform for bioconversion of mixed-biomass hydrolysate feedstocks [8, 9]. *R.* *toruloides* has been engineered to make a number of chemically diverse bioproducts including nonribosomal peptides such as indigoidine [10], second-generation biofuels such as bisabolene [11], and industrial chemicals such as fatty alcohols [12].

While *R.* *toruloides* is naturally competent to catabolize pentose sugars, both specific growth rate and biomass yield are lower for xylose than glucose—approximately 0.14 1/h on xylose vs 0.32 1/h on glucose [13] and 0.35 g/g on xylose vs 0.45 g/g on glucose [13], respectively. Furthermore, in mixed-sugar biomass hydrolysates, xylose utilization only occurs after glucose has been mostly consumed, and a transitional lag phase is often observed [11, 14]. Though this diauxic sugar utilization is little studied in *R.* *toruloides*, it is a common finding across diverse fungi, generally believed a consequence of both competition between glucose and xylose for transport into the cell [15], and carbon catabolite repression [16] of alternative catabolism genes. Improving any of these quantities—yield or specific growth rate on xylose, rates of sugar co-transport, or accelerating the transition to xylose utilization—would enhance the overall efficiency of bioproduct conversion from renewable biomass. Up to this point, little is known about regulation of pentose catabolism in *R.* *toruloides*, or in any basidiomycete yeast. Such regulatory proteins would be attractive metabolic engineering targets for basidiomycetes cultivated on biomass hydrolysates.

In a recent study, we employed high-throughput fitness profiling in combination with transcriptomic and proteomic profiling to build an improved model of *R.* *toruloides* carbon metabolism [4]. We observed that mutations in two genes with likely regulatory functions altered strain fitness on xylose and other pentose utilization intermediates. Mutations in the predicted transcription factor RTO4_12978 (Uniprot ID A0A0K3CFC7) greatly decreased fitness on d-xylose, l-arabinose, l-lyxose, and several pentose metabolism intermediates. RTO4_12978 is a member of the fungal-specific zinc binuclear cluster family of transcription factors. Zinc binuclear cluster transcription factors are known to regulate a wide range of cellular processes, but many have been characterized as regulators of alternative carbon catabolism [17], including several known regulators of catabolism of pentose sugars or pentose-containing polysaccharides in fungi. In our fitness assays, no other transcription factors were absolutely required for growth on pentose sugars and alcohols, thus RTO4_12978 was a prime candidate for a major regulator of pentose catabolism. We provisionally named RTO4_12978 Pnt1 for pentose catabolism regulator 1.

**Fig. 1:**
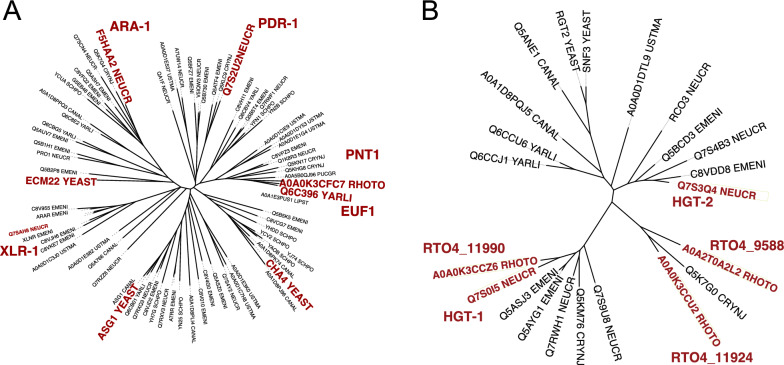
**A** Phylogenetic tree of *R.* *toruloides* Pnt1 and the *N.* *crassa* alternative carbon regulatory transcription factors Ara-1, Pdr-1, and Xlr-1. Sequences shown here were assembled from separate BLAST searches for each of these proteins to a database of seven model fungi (see “Methods”). Highlighted proteins have experimentally informed functions discussed in the text. **B** Phylogenetic tree of RTO4_11990, RTO4_9588, and RTO4_11924 with *S.* *cerevisiae* Snf3, *N.* *crassa* HGT-1, *N.* *crassa* HGT-2 and other homologs from seven model fungi

A phylogenetic tree of the most closely related sequences to Pnt1 and the zinc binuclear cluster transcription factors Xlr-1, Ara-1, and Pdr-1 (known regulators of pentose metabolism and degradation of pentose-containing polymers in *Neurospora* *crassa* [18]) is shown in Fig. 1A. Transcription factors that are more closely related to Pnt1 than the known pentose regulators include the *Saccharomyces* *cerevisiae* transcription factors Ecm22, Asg1, and Cha4—regulators of sterol biosynthesis [19], fatty acid metabolism [20], and amino acid catabolism [21], respectively. The most closely related protein with any available functional data, however, is Euf1 from *Yarrowia* *lipolytica* (UniProt ID YALI0F01562p), a regulator of erythritol catabolism [22]. Though it is believed that erythritol catabolism ultimately occurs through the pentose phosphate pathway [23], no studies exist investigating Euf1’s role in pentose catabolism. However, the authors did note an accumulation of arabitol and mannitol in the media of ∆Euf1 mutants. In a separate study, a point mutation in YALI0F01562p was identified in a strain adapted for better growth on xylose [24], but that mutation was one of many, and the authors could find no closely related proteins with known regulation of alternative carbon metabolism, so it was not investigated further. In retrospect, the authors may have discovered an important regulator of pentose catabolism in *Y.* *lipolytica* but were dissuaded from further study on its function by the disparate known functions of all characterized homologs at that time.

The Kim et al. study also found that mutations in the predicted transmembrane protein RTO4_11990 (UniProt ID A0A0K3CCZ6) increased fitness on xylose, but not on arabinose, lyxose, or pentose catabolic intermediates. RTO4_11990 has high sequence similarity to *Saccharomyces* *cerevisiae* glucose sensors Snf3/Rgt2, which work in tandem as low/high affinity glucose sensors [25]. A phylogenetic tree of Protein ID RTO4_11990 and closely related proteins from seven model fungi is presented in Fig. 1B. The most closely related characterized protein to RTO4_11990 in this analysis is *N*. *crassa* HGT-1. Together with a close homolog HGT-2, HGT-1 forms a high/low affinity glucose transport and signaling system with a role in carbon catabolite repression [26]. The most closely related *R.* *toruloides* proteins to HGT-2 are RTO4_11924 and RTO4_9588 (47% and 48% identity to HGT-2, respectively), though they are not obviously orthologous.

In this work, we explore involvement of Pnt1 and three HGT-1/2 homologs in regulation of xylose metabolism with overexpression and deletion mutants, respectively. We demonstrate that Pnt1 is, indeed, a major regulator of pentose catabolism in *R.* *toruloides.* We confirm that RTO4_11990 plays some role in regulation of pentose metabolism, but that function is not strictly analogous to that of *N.* *crassa* HGT-1/HGT-2. Whether or not RTO4_11990 truly acts as a tranceptor and which molecule it binds remains unproven. To show that modulating pentose catabolic enzyme expression can improve conversion of xylose to a commercially relevant bioproduct many enzymatic steps removed from pentose assimilation, we overexpress Pnt1 in a fatty alcohol production strain. Finally, we explore the Pnt1 regulon with proteomic analysis of Pnt1 overexpression and deletion mutants in the presence of xylose.

## Results

**Fig. 2:**
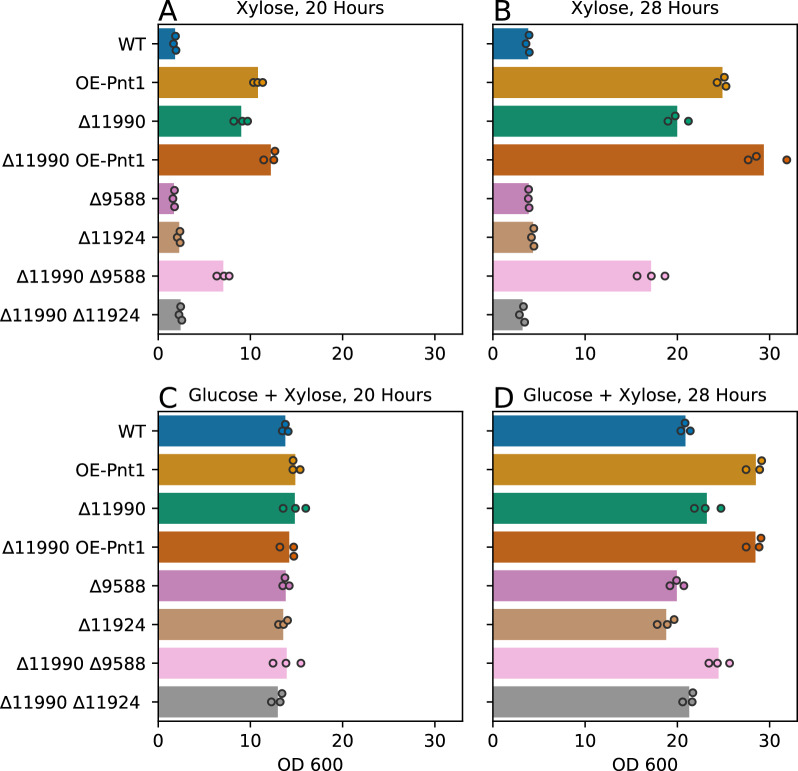
Growth of *R.* *toruloides* strains with overexpression of Pnt1 and/or deletion of HGT-1/HGT-2 homologs on xylose and mixed glucose plus xylose media. **A**, **B** 40 g/L xylose; 20 h and 28 h, respectively. **C**, **D** 20 g/L xylose, 20 g/L glucose; 20 h and 28 h, respectively

**Fig. 3:**
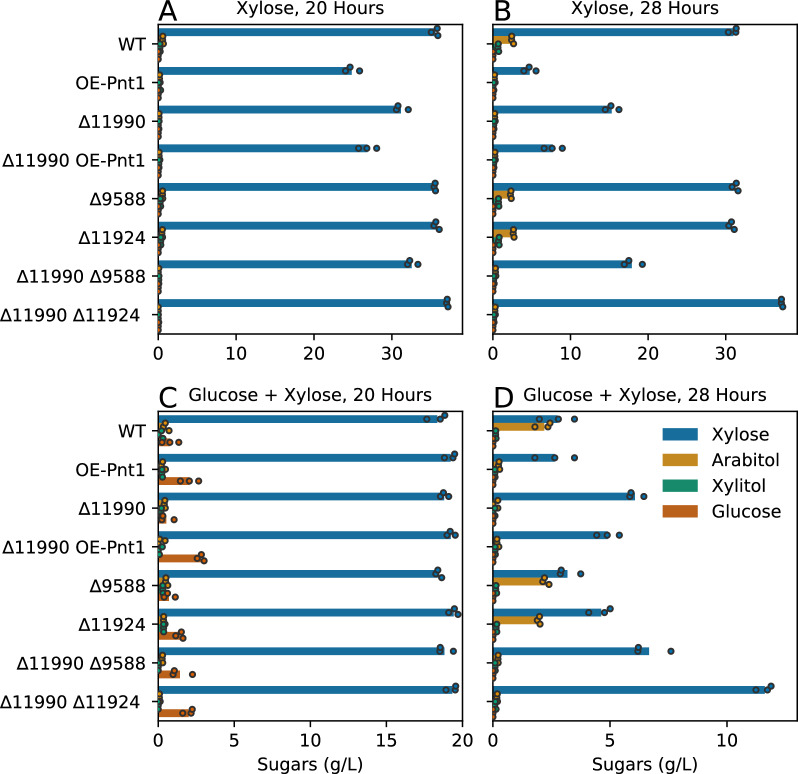
Remaining sugars and sugar alcohols in culture medium of *R.* *toruloides* strains with overexpression of Pnt1 and/or deletion of HGT-1/HGT-2 homologs grown on xylose and mixed glucose plus xylose media. **A** 40 g/L xylose at 20 h. **B** 40 g/L xylose at 28 h. **C** 20 g/L xylose plus20 g/L glucose at 20 h. **D** 20 g/L xylose plus 20 g/L glucose at 28 h

To confirm Pnt1 xylose catabolism regulation and improve xylose utilization in *R.* *toruloides,* we constructed a Pnt1 overexpression strain by random integration of the Pnt1 coding sequence under control of the *Rhodotorula* *graminis* Tef1 promoter and the *R.* *toruloides* Nos terminator [27], introduced by *Agrobacterium* *tumefaciens*-mediated transformation (see “Methods”). The *R.* *graminis* Tef1 promoter was selected as a strong promoter with sufficient sequence divergence from the native Tef1 locus to reduce disruptive integration of the overexpression construct at that locus. When cultured on xylose as the sole carbon source, OE-Pnt1 mutants grew faster on xylose than the parent strain IFO 0880 (WT), reaching 24.9 vs 3.8 OD units in 28 h (pVal 1.6e−7) (Fig. 2A, B). During this period the OE-Pnt1 strain consumed more xylose than WT (35.3 vs 9.0 g/L, pVal 6.2e−7) (Fig. 3A, B). When cultured on media containing both glucose and xylose, the OE-Pnt1 and WT strains grew similarly for the first 20 h, then as glucose was consumed and both strains shifted to xylose utilization, OE-Pnt1 again outpaced WT (28.5 vs 20.9 OD units at 28 h, pVal 0.0001, Fig. 2D). At 28 h the OE-Pnt1 and WT strains had consumed similar amounts of xylose (17.4 vs 17.2 g/L), but the WT strain had accumulated significant arabitol (2.2 g/L) while the OE-Pnt1 strain was near the limit of detection for our HPLC analysis (around or less than 0.3 g/L, pVal vs WT 0.0003) (Fig. 3D), thus net utilization of pentose sugar (xylose imported and fully metabolized, as opposed to exported as sugar alcohol) was greater for OE-Pnt1.

We then grew OE-Pnt1 and WT on 40 g/L xylose and 40 g/L glucose plus 40 g/L xylose media for a longer period and monitored growth, sugar consumption, and sugar alcohol production (Additional file 3: Fig. S1). The OE-Pnt1 mutant accumulated less sugar alcohol in the media than WT in both conditions, especially at the end of the experiment, with 0.3 g/L vs 8.1 g/L arabitol (pVal 1.6e−5) on xylose and 0.7 g/L vs 2.5 g/L arabitol (pVal 0.001) on glucose plus xylose, at 45 h. Assuming a linear relationship between optical density and biomass (approximately 0.5 g/L per OD unit) and assuming that growth during the last phase of xylose consumption (21–29 h for OE-Pnt1 on xylose, 29–45 h all other conditions) is dominated by xylose catabolism (not yeast extract or glucose), net biomass yield (g biomass / g xylose fully metabolized) was equivalent between OE-Pnt1 and WT on xylose (0.29 g/g and 0.30 g/g, pVal 0.4) and on glucose plus xylose (0.22 g/g and 0.19 g/g, pVal 0.1).

In a high-resolution time course of OE-Pnt1 and WT growth on 40 g/L xylose (Additional file 3: Fig. S2), OE-Pnt1 had a faster initial growth rate and reached a higher maximum specific growth more quickly (0.17 at 30 h vs 0.14 at 50 h, pVal 5.8e−5). However, the relationship between absorbance measured from this instrument (Biolector Pro) and true biomass can change with cell morphology and culture density. Thus, caution should be exercised when interpreting the different apparent maximum growth rates as they occurred at different points along the growth curve. The most robust result is the approximately twofold difference in specific growth of the strains between 10 and 30 h. In this experiment the WT strain had a higher final absorbance than OE-Pnt1 (89.1 vs 70.6 AU, pVal 5.5e−4), which may be reflective of a higher biomass yield in this condition, or morphology differences between the strains. This experiment was conducted with ammonium sulfate as the nitrogen source (as opposed to yeast extract for previous experiments) which would suggest that any consistent biomass yield differences between these strains may be dependent on growth conditions.

These results are consistent with increased expression of xylose catabolic enzymes in OE-Pnt1, increasing initial growth rate when transitioning to xylose metabolism and relieving metabolic bottlenecks within the early stages of pentose catabolism that cause accumulation of xylitol and arabitol in the WT strain However, when glucose is present in high concentrations, a combination of competitive transport and carbon catabolite repression still prevent xylose co-utilization in the OE-Pnt1 mutant.

To test if the three HGT-1/2 homologs (RTO4_11990, RTO4_9588, and RTO4_11924) contribute to glucose-induced carbon catabolite repression of xylose catabolism in *R.* *toruloides,* we constructed single- and double-deletion mutants for the putative transceptors. None of these mutants grew dramatically faster or utilized xylose more quickly in the presence of glucose (Figs. 2C, D, 3C, D). The ∆11990 and ∆11990 ∆9588 mutants reached slightly higher density than WT at 28 h (23.2 and 24.5 OD units vs 20.9 OD units, pVals 0.03 and 0.004, respectively), though they also consumed less xylose (13.9 and 13.2 g/L vs 17.2 g/L, pVals 0.001 and 0.002, respectively), and accumulated less sugar alcohol (0.1 and 0.2 g/L vs 2.2 g/L, pVal ~ 0.0003). Unexpectedly the ∆11990 mutant had a stronger phenotype vs WT in the absence of glucose (reaching 20.0 vs 3.8 OD units at 28 h on xylose, pVal 8.2e−6). The double mutant ∆11990 OE-Pnt1 was the fastest growing mutant of the set, outpacing the OE-Pnt1 mutant (reaching 29.4 vs 24.9 OD units at 28 h on xylose, pVal 0.01). The double mutant consumed slightly less sugar than OE-Pnt1, (32.3–35.3 g/L, pVal 0.01). The ∆9588 and ∆11924 mutants did not behave differently than WT in these experiments. The ∆9588 ∆11990 double mutant grew similarly to ∆11990. The ∆11924 ∆11990 mutant had a general growth defect that complicates analysis, and grew less quickly on xylose than the ∆11990 strain did.

These data are not consistent with a conserved role for the HGT-1/2 homologs in glucose-specific carbon catabolite repression of the xylose utilization pathway. Rather, these data and our previous high-throughput data are most consistent with a role for RTO4_11990 in glucose-independent modulation of pentose catabolism, possibly in response to xylose itself, a phenomenon reported for *S.* *cerevisiae* Rgt2/Snf3 [28]. The functions of RTO4_9588 and RTO4_11924 remain unclear.

Given the OE-Pnt1 and ∆11990 mutant’s faster growth when transferred to xylose or transitioning from glucose to xylose utilization in mixed sugars, we hypothesized that these mutations could improve the overall rate of bioproduct conversion from xylose and xylose-containing hydrolysates. To test this, we deleted RTO4_11990 and overexpressed Pnt1 in a strain engineered to produce fatty alcohols (FOHs). We created the ∆11990 OE-Pnt1 FOH production strain by replacing the RTO4_11990 locus with the Pnt1 overexpression construct. We also isolated three independent strains arising from random integrations of the Pnt1 overexpression construct in the parental genome (OE-Pnt1 R1-3).

**Fig. 4:**
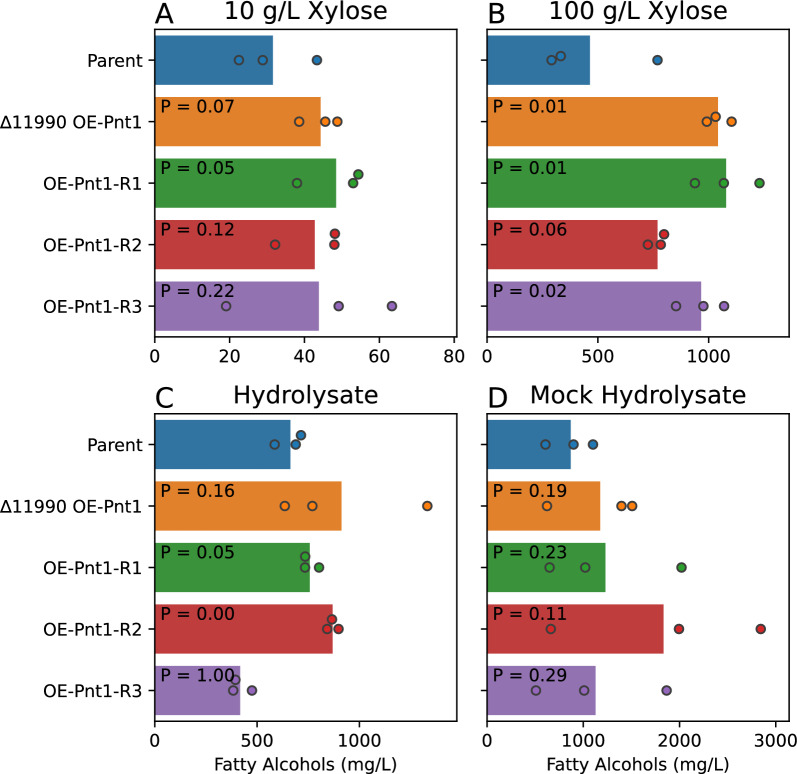
Fatty alcohol production in strains overexpressing Pnt1 from the Tef1 promoter, either integrated at the RTO4_11990 locus, or randomly integrated into the genome. **A** 10 g/L xylose YNB medium. **B** 100 g/L xylose YNB medium (**C**) Corn stover hydrolysate: approximately 75 g/L glucose, 40 g/L xylose. **D** Mock hydrolysate with 75 g/L glucose, 40 g/L xylose in YNB medium. P-values from a student’s T-test vs the parent strain are shown for each strain

In order to test relative rates of xylose conversion to fatty alcohol we grew the mutants and the parental fatty alcohol producing strain in a panel of media conditions. The media formulations tested were 10 g/L xylose, 100 g/L xylose, corn stover hydrolysate (comprising approximately 75 g/L glucose, 40 g/L xylose, 5 g/L arabinose and smaller amounts of other plant biomass components (see “Methods” [29]), and mock hydrolysate with similar sugar concentrations to the true hydrolysate (see “Methods”). After 5 days of growth and fatty alcohol production, the cultures were stopped, sampled, and measured for fatty alcohol by gas chromatography (Fig. 4). We chose a 5-day culture period to sample before the end of xylose consumption in most conditions, so that a single timepoint would capture relative rates of product formation (as opposed to maximum titer or yield). We tested the 5-day samples for remaining xylose with a commercial enzymatic assay and verified that substantial xylose remained for all conditions except for the 10 g/L xylose cultures (Additional file 3: Fig. S3). Thus the relative performance of the mutants may be underestimated in the 10 g/L xylose condition as the parental strain had time to catch up to the mutants. Differential growth rates between the parent and OE-Pnt1 strain confound interpretation of specific production rates (g product/g cells/h) from these data.

The ∆11990 OE-Pnt1 strain accumulated approximately 120% more fatty alcohol than the parent strain in the 100 g/L xylose condition (1040 mg/L vs 460 mg/L, pVal 0.01), and 30–40% more fatty alcohols in the other conditions, though high culture-to-culture variability limited the statistical confidence of the apparent increase on hydrolysate (910 mg/L vs 660 mg/L, pVal 0.16) and mock hydrolysate media (1180 mg/L vs 870 mg/L, pVal 0.19) (Fig. 4). Fatty alcohol production was generally similar in the ∆11990 OE-Pnt1 strain and the random integration strains, though in each condition a few biological replicates of the random integration strains tended to have outlier fatty alcohol concentrations. These differences between random integration strains could be the result of variable Pnt1 expression due to different copy number insertions, or due to background mutations in the parental genome. Indeed, in our construction of the OE-Pnt1 strain in the WT background, we isolated transformants with a range of robustness of antibiotic resistance and different growth rates on xylose, consistent with a range of different copy number integrations (see “Methods”). In cultures with extreme outlier fatty alcohol concentrations, e.g., OE-Pnt1-R2 on mock hydrolysate, higher fatty alcohol production correlated with greater xylose consumption. Thus, the differences observed are likely attributable to variations in initial inoculation (a possibility if random insertions result in subtle morphology defects and thus a differing relationship between optical density and biomass) or stochastic variation in Pnt1 expression (a possibility if multiple insertions are present in differing genomic locations). When considered as a group, the random insertion mutants tended to average similar fatty alcohol production to the single copy ∆11990 OE-Pnt1 strain. These results confirmed that improved growth dynamics on xylose from Pnt1 overexpression can translate into a faster rate of bioproduct conversion, under the right conditions, but failed to demonstrate any unambiguous additive benefit for deletion RTO4_11990 in this context.

Given the significant improvement in xylose conversion to fatty alcohol that we achieved by overexpressing Pnt1, a systematic characterization of the Pnt1 regulon was warranted. Therefore, we compared global protein abundance in WT, OE-Pnt1, and a Pnt1 deletion strain (∆Pnt1). To best capture the full regulon of Pnt1 under xylose utilization conditions and minimize possible confounding effects of carbon catabolite repression, while still supporting sufficient growth of the ∆Pnt1 strain, we sampled cultures in exponential phase on medium with 40 g/L xylose as the sole carbon source and on medium with 40 g/L xylose plus 40 g/L glycerol as a neutral carbon source with respect to xylose [30]. To minimize physiological differences at different growth phases, we sampled cultures during exponential phase at constant OD, as opposed to at a constant time. As these were batch cultures grown without pH or dissolved oxygen control, some differences in these parameters may have accumulated at the time of sampling. We mapped 46,486 unique peptide sequences to 4582 *R.* *toruloides* proteins. For each protein we calculated a filtered median peptide intensity, reflective of protein abundance relative to total protein in the sample. Overall consistency between biological replicates was excellent across the experiment, with clear distinction between all treatment groups in a principal component analysis of protein intensities (Additional file 3: Fig. S4). All peptide intensities and statistical analyses are reported in Additional file 1.

**Fig. 5:**
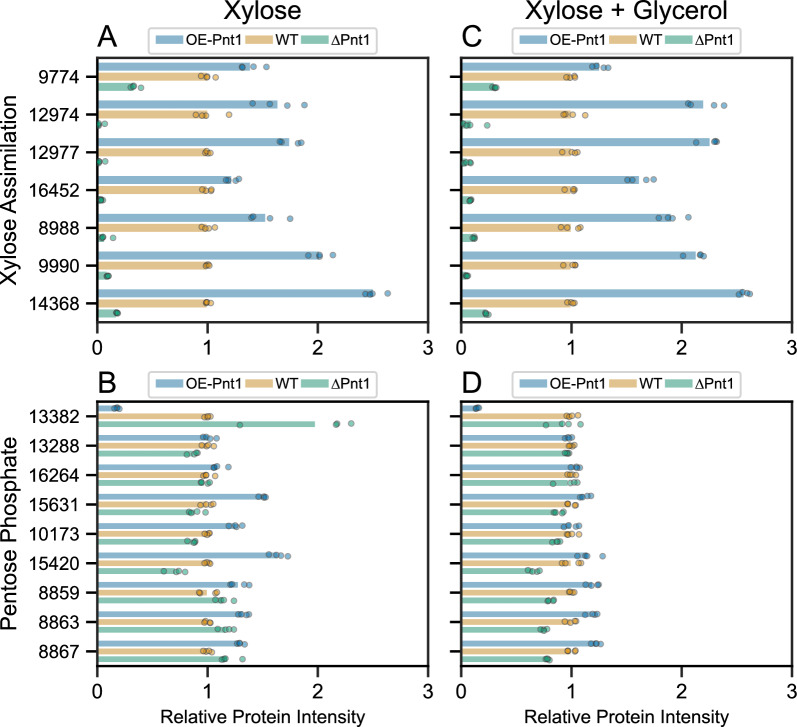
Relative intensities for proteins with roles in xylose assimilation (**A**, **C**) or the pentose phosphate pathway (**B**, **D**) in strains OE-Pnt1, ∆Pnt1, and WT (IFO 0880). Circles indicate protein intensities for each of four biological replicates for each condition. Relative intensity for each sample is normalized to the average intensity for WT in the same condition. Enzymatic activities of these proteins in the *R.* *toruloides* metabolic model are shown in Fig. 6

**Fig. 6:**
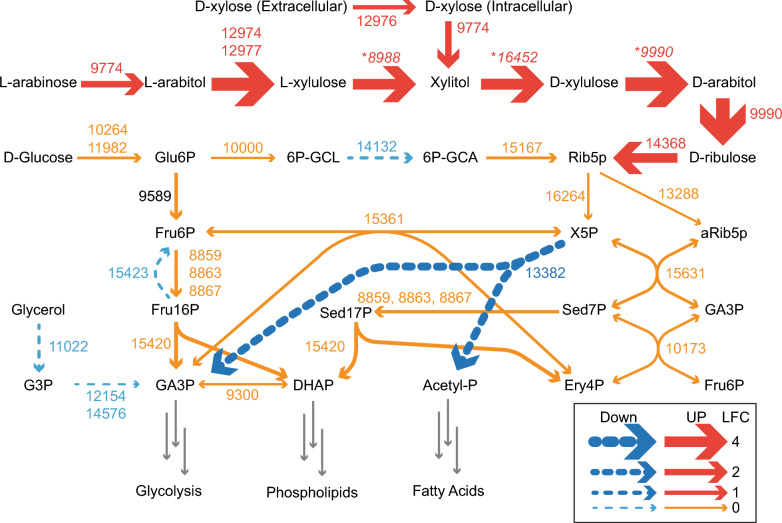
The pentose assimilation and pentose phosphate pathways of *R.* *toruloides* with abundance changes in Pnt1 mutants. Arrow size is proportional to the log_2_-fold change (LFC) between OE-Pnt1 and ∆Pnt1 strains on xylose plus glycerol, except for RTO4_12976 which was undetected in ∆Pnt1. In cases where multiple proteins are assigned to a reaction, the lowest magnitude fold change is represented. *XXXX = Protein for which high throughput fitness data confirmed function in the pathway but was inconclusive as to which enzymatic reaction(s) are mediated. Metabolite abbreviations: *Glu6P* d-glucose-6-phosphate, *6P-GCL* 6-phospho-d-glucono-1,5-lactone, *6P-GCA* 6-phospho-d-gluconate, *Rib5p* d-ribulose 5-phosphate, *aRib5p* alpha-d-ribulose-5-phosphate, *Fru6P* d-fructose-6-phosphate, *X5P* xylulose-5-phosphate, *Fru16P* d-fructose-1-6-bisphosphate, *Sed17P* sedoheptulose 1,7-bisphosphate, *Sed7P* sedoheptulose 7-phosphate, *GA3P* glyceraldehyde 3-phosphate, *DHAP* dihydroxyacetone phosphate, *Acetyl-P* acetyl phosphate, *Ery4P* d-erythrose 4-phosphate, *G3P* glycerol-3-phosphate

The key proteins in our metabolic model for pentose assimilation, for which mutants had significant fitness defects in high-throughput RB-TDNAseq assays on pentose sugars and alcohols [4], were amongst the most strongly Pnt1-induced proteins across the experiment (Fig. 5A, B). For most enzymes in the pentose phosphate pathway, we observed modest changes in abundance across strains, with smaller differences in glycerol-supplemented media (Fig. 5C, D). These results are more consistent with a response to faster growth and, presumably, a higher cellular energy state in the OE-Pnt1 strains on xylose and a smaller growth advantage in glycerol-supplemented media, as opposed to a direct induction of expression by Pnt1 in response to xylose. RTO4_13382 (xylulose phosphoketolase), however, was much less abundant in OE-Pnt1 vs WT in both media conditions, and was previously shown to be less abundant in WT on xylose vs glucose [4]. While RTO4_13382 was significantly more abundant in ∆Pnt1 vs WT on xylose, abundances in ∆Pnt1 and WT were similar upon glycerol supplementation. It should be noted that glyceraldehyde-3-phosphate is a direct product of xylulose phosphoketolase and as such, in the presence of abundant glycerol, feedback regulation could confound effects of Pnt1 regulation of this protein’s expression. Effects of Pnt1 mutations across pentose assimilation and the pentose phosphate pathway are summarized in Fig. 6, and across the *R.* *toruloides* metabolic model in (visualized with Escher [31]) Additional file 3: Fig. S5. Note that the metabolic models of *R.* *toruloides* pentose catabolism used in these figures come from the high-throughput studies of Kim et al. [4]. The details of this pathway are under active investigation and may be updated in future publications, especially for the proteins marked with an asterisk in Fig. 6.

The WT, ∆Pnt1, and OE-Pnt1 strains had different growth rates on xylose-containing media, even with glycerol present. Doubling time was over 7 h for ∆Pnt1 on xylose plus glycerol and optical density increased less than 10% after 30 h on xylose media, whereas doubling time was approximately 4 h for OE-Pnt1 in both media conditions, and 6 to 10 h for WT. As a result, large-scale proteomic differences were present across conditions. Over 2800 protein intensities were significantly different between WT and a Pnt1 mutant in at least one condition. We thus limited our analysis to proteins with a pattern of abundance consistent with Pnt1 induction or repression by xylose: a statistically significant (multiple-hypothesis adjusted P-values < 0.1) two-fold or greater change in abundance between ∆Pnt1 and OE-Pnt1 strains in both conditions, with an intermediate abundance in WT. A hierarchical clustering analysis of the 147 proteins that met these criteria is presented in Additional file 3: Fig. S6. A complete table of protein intensity scores and statistical analysis used to generate this subset of differentially regulated proteins is included in Additional file 1.

Cross referencing differentially abundant proteins in Pnt1 mutants with proteomic data from WT *R.* *toruloides* grown on multiple carbon sources as reported in [4], we noted that only 14 proteins had abundance patterns in Pnt1 mutants consistent with positive regulation of expression by Pnt1 and also were more abundant in WT on xylose than WT on glucose in the previously published data. Similarly, only 8 proteins had abundance patterns consistent with repression by Pnt1 and lower abundance in WT on xylose than WT on glucose. These proteins, constituting the Pnt1-dependent xylose regulon, are listed in Table 1. The rest of the 147 proteins with differential abundance across the Pnt1 mutant strains had abundance patterns across the two datasets more consistent with regulation by cellular energy state than by a direct Pnt1-mediated response to xylose. For example, RTO4_8709 adenylylsulfate kinase, was over fourfold more abundant in OE-Pnt1 than ∆Pnt1 on xylose but also over fivefold more abundant in WT on glucose than WT on xylose (see Additional file 1 for a comparison of relative abundances across both data sets). The proteins listed in Table 1 which are not already accounted for in the *R.* *toruloides* metabolic model (Fig. 6) are good candidates for further examination in the context of pentose catabolism and xylose conversion to bioproducts.

**Table 1:** The Pnt1 regulon

Protein	LFC	Description
12974	6.6	l-arabitol dehydrogenase
12977	6.3	l-arabitol dehydrogenase
16452	5.1	Predicted xylitol dehydrogenase
8504	4.9	Aldose 1-epimerase
8988	4.6	Predicted l-xylulose reductase
9990	4.4	Predicted d-arabitol dehydrogenase
14368	3.8	Ribulokinase
16635	3.5	*p*-Coumarate-CoA ligase
9798	3.2	RNA-binding polyadenylation factor
10421	2.2	Similar to 4-hydroxymuconic semialdehyde dehydrogenase
12248	2.1	NADP(+)-dependent glutamate dehydrogenase
9774	2.0	Xylose reductase
13328	1.3	d-lactate dehydrogenase
12976	(+)	Similar to xylose transporters
12917	(−)	Protein of unknown function
11642	− 1.9	Similar to lipid/aromatic oxidoreductases
13088	− 2.1	Metalloendoprotease
11085	− 2.2	Similar to nitronate monooxygenase
14597	− 2.2	Acetyl-CoA synthetase
9868	− 2.7	TPSO/MBR family protein
9856	− 3.0	NAD(+) specific glutamate dehydrogenase
15678	− 3.2	Protein of unknown function

LFC: Log-Fold Change of OE-Pnt1 vs ∆Pnt1 on xylose. LFC could not be calculated for RTO4_12976 and RTO4_12917 as they were not detected in the ∆Pnt1 and OE-Pnt1 strain, respectively

## Discussion

Our results confirm that Pnt1 is a major regulator of pentose catabolism in *R.* *toruloides.* Given our data and established mechanisms for other zinc binuclear cluster transcription factors, Pnt1 most likely acts primarily as a transcriptional activator of gene expression. Direct binding and transcriptomic studies would be required to conclusively demonstrate that mechanism, however.

Our identification of Pnt1 illustrates the value of unbiased high-throughput methods to uncover gene function, despite the large corpus of gene functions characterized in diverse model systems. At the outset of these experiments, an extremely thorough manual review of all known transcriptional regulators of alternative catabolism may well have identified RTO4_12978 as a potential protein of interest due to its similarity to a reported regulator of erythritol catabolism in a distantly related yeast. However, the preponderance of closely related sequences with known or inferred roles in unrelated processes greatly obfuscates any a priori prediction of RTO4_12978’s function. Implementing functional high-throughput screening methods directly in strains of interest opens the opportunity to better leverage the unique aspects of those strains’ biology for biotechnological applications.

Only one protein not accounted for in our existing metabolic model for pentose catabolism [4] had comparable magnitude of abundance changes to the major known enzymes of pentose assimilation: RTO4_8504, similar to aldose-1 epimerases. Co-regulation of RTO4_8504 with the rest of the xylose assimilation pathway implies that in *R.* *toruloides’* natural environment, the spontaneous interconversion between alpha and beta anomers of a pentose sugar may become rate limiting in some cases, even though RTO4_8504 mutations had no significant fitness defect when grown on any the pentose sugars we tested in our high-throughput assay [4].

A few proteins had more modest, but consistent and statistically significant changes in abundance in Pnt1 mutants. These included RTO4_16635, p-coumarate CoA ligase [4] and RTO4_10421, an aldehyde dehydrogenase with some similarity to p-cumic aldehyde dehydrogenase cymC from *Pseudomonas* *putida* [32]. Most likely there is an adaptive advantage for *R.* *toruloides* to poise rate-limiting steps for catabolism of aromatic derivatives of lignin when consuming xylose, which will reliably be found in the presence of decaying plant matter. RTO4_13328, orthologous to *S.* *cerevisiae* D-lactate dehydrogenase DLD1 may play some role in removing 2-hydroxyglutarate accumulation under stress [33] or, given the promiscuity of many oxidoreductases involved in pentose metabolism, it may play some more direct role in pentose assimilation. RNA-binding polyadenylation factor RTO4_9798 may have a general role in stress response [34], or a specific role in regulation of expression of pentose assimilation proteins.

In *S.* *cerevisiae* engineered to improve xylose fermentation, it was advantageous to delete the major NADPH-dependent glutamate dehydrogenase (GDH) and overexpress an NADH-dependent GDH to compensate, reducing competition for NADPH required for xylose reductase [35]. We found the ortholog of the *S.* *cerevisiae* NADPH-dependent GDH (RTO4_12248) abundance was correlated with Pnt1 abundance, while the ortholog of the *S.* *cerevisiae* NADH-dependent GDH (RTO4_9856) was inversely correlated. Direct binding studies would be required to distinguish if these effects result from direct action of Pnt1 or as a consequence of altered NADPH/NADH ratios in these mutants. Regardless of the specific mechanism involved, *R.* *toruloides’* native regulation of GDH expression on xylose apparently favors a shift towards more of the NADPH-dependent enzyme when pentose catabolism is upregulated by Pnt1. This shift will likely need to be overridden in *R.* *toruloides* strains optimized for growth on xylose in order to reduce competition for NADPH.

Ultimately, our goal in exploring *R.* *toruloides* native xylose metabolism is to enable improved conversion of xylose to bioproducts. Through manipulation of Pnt1 expression, we found a convenient tool to increase expression of major enzymes converting xylose to pentose phosphate pathway intermediates. This coordinated upregulation was sufficient to increase the initial specific growth rate twofold and maximum specific growth by 18% when OE-Pnt1 was inoculated in medium with xylose as the sole carbon source, and reduce the transitional lag when shifting from glucose consumption to xylose consumption in medium containing both sugars.

These improvements in the dynamics of growth and xylose utilization translated into a 120% increase in the overall rate of fatty alcohol production in a 5-day batch culture on 100 g/L xylose. The corresponding rate increase in fatty alcohol production was more modest (~ 35%) on biomass hydrolysate medium containing a mix of glucose and pentose sugars, commensurate with a smaller increase in overall growth rates of OE-Pnt1 on media with both glucose and xylose. The fatty alcohol concentrations achieved in this experiment were comparable to reported titers from other oleaginous engineered for fatty alcohol production cultivated on xylose in bioreactor systems (~ 1 g/L) [36, 37], but substantially below recently reported titers for highly engineered fatty alcohol strains grown on glucose in optimized bioreactor studies (as high as 8 g/L) [38–41]. Our objective in these experiments was to demonstrate that manipulation of pentose catabolite enzyme expression through regulatory proteins can improve the rate of bioproduct conversion from pentose-rich biomass hydrolysates to higher value products under certain conditions. Further strain engineering and process development will be needed to achieve higher titers and yields. The ultimate impact of such manipulations will be highly dependent on the details of hydrolysate composition, production conditions, and final product, but regulation of alternative carbon catabolism should be considered in any multifactorial engineering strategy for bioconversion of complex substrates.

As we identified RTO4_11990—a close relative of putative glucose transceptors participating in carbon catabolite repression signaling [26]—in our high throughput fitness assays on xylose, a natural extension of our manipulation of Pnt1 expression was to delete RTO4_11990 in an attempt to reduce the effects of carbon catabolite repression. Surprisingly the effects of deleting RTO4_11990 were very similar to those of Pnt1 overexpression, with faster initial growth on xylose and a slightly faster transition to xylose consumption as glucose was exhausted in cultures with both glucose and xylose, but with no apparent increase in the co-utilization of xylose and glucose. We therefore conclude that RTO4_11990 may function as a tranceptor regulating xylose catabolism, but if it does, then glucose is not the molecule that it primarily interacts with. Deletions for two more homologs of reported glucose transceptors involved in carbon catabolite repression in other fungi also did not increase the rate of xylose co-utilization with glucose in isolation or in combination with deletion of RTO4_11990. We cannot rule out some function in glucose sensing of one or more of these putative transceptors in cooperation with additional proteins, but have no evidence to that effect at this time. Given the need to optimize production of bioproducts from mixed substrates derived from lignocellulosic biomass, a deeper exploration of the key factors mediating carbon catabolite repression in *R.* *toruloides* is warranted.

## Conclusions

The zinc binuclear cluster transcription factor Pnt1 is a major regulator of pentose catabolism in *R.* *toruloides.* The Pnt1 regulon in response to xylose is dominated by proteins with known or suspected roles in pentose utilization in *R.* *toruloides*, reported to impact pentose catabolism in other species, or with predicted function that could relate to catabolism of aromatic compounds associated with decaying plant matter. Overexpression of Pnt1 is sufficient to increase the abundance of major enzymes in the pentose catabolism pathway and increase the rate of xylose utilization when transitioning from other carbon sources. This increased rate of xylose utilization translated to an increased overall rate of bioproduct production in some xylose-rich conditions. Manipulation of Pnt1 expression is an important new tool to optimize the conversion of pentose-rich biomass to higher value bioproducts in *R.* *toruloides*.

## Methods

### Phylogenetic analysis

The protein sequences were retrieved from Uniprot and searched against a local database of nine fungal proteomes also retrieved from Uniprot [42]: *Candida* *albicans* (UP000000559), *Cryptococcus* *neoformans* (UP000002149), *Emericella* *nidulans* (UP000000560), *Neurospora* *crassa* (UP000001805), *Saccharomyces* *Cerevisiae* (UP000002311), *Schizosaccharomyces* *pombe* (UP000002485), *Rhodosporidium* *toruloides* (UP000239560), *Ustilago* *maydis* (UP000000561), and *Yarrowia* *lipolytica* (UP000001300). Significant matches (E-value < 0.01) were aligned with MAFFT [43]. Phylogenetic trees were constructed with FastTree [44]. Trees were visualized with iTol [45].

### Strains and sequences

*Rhodosporidium* *toruloides* (a.k.a. *Rhodotorula* *toruloides,* a.k.a *Rhodotorula* *graciis*) strain IFO 0880 (a.k.a NBRC 0880) was obtained from the Biological Resource Center, NITE (NBRC), Japan. All strains named in this work are available to order through the Agile BioFoundry parts registry at https://public-registry.agilebiofoundry.org. The registry website also hosts all applicable plasmid sequences. Applicable strains and plasmid sequences are listed by figure in Additional file 2. Protein identification numbers used in this manuscript are from the *R.* *toruloides* genome version 4, available on Mycocosm, the US Department of Energy Joint Genome Institute fungal genome repository [46]. Selection markers used in *R.* *toruloides* were hygromycin and G418 resistance cassettes using the *R.* *toruloides* Tub2 promoter and terminator (see sequences on the Agile BioFoundry parts registry). Some selectable markers had a C-terminal fused sequence of thymidine kinase from herpes simplex virus, a useful construct for counter-selection and marker recovery [47], though no markers were recycled in this study.

For strains constructed by homologous recombination (e.g., full-deletion mutants), the parental strain was either a deletion mutant of the non-homologous end-joining factor Ku70 [48] or wild type. Homologous recombination and non-homologous end-joining (i.e., for generating randomly integrated mutants) was achieved by transforming *R.* *toruloides* with linearized plasmid by a lithium acetate transformation protocol as described in [49], or TDNA insertion by *Agrobacterium* *tumefaciens*-mediated transformation as described in [50]. Strain construction methods are listed for each strain in Additional file 2. For all deletion mutants, successful deletion was confirmed by diagnostic PCR at the altered locus. For targeted insertion of OE-Pnt1 at the RTO4_11990 locus, insertion was confirmed by diagnostic PCR followed by sequence verification of the inserted sequence. For construction of the OE-Pnt1 strain by random insertion, 24 randomly selected transformants were screened for growth in liquid culture with 600 µg/mL of the selective agent G418 (100 µg/mL is a common concentration for routine selection with G418). Significant variation was observed in growth rates amongst transformants, likely a consequence of different levels of expression of the selective marker due to local chromatin structure, number of integrations, and some rate of incomplete partial TDNA integrations. The three fastest growing strains were tested for growth on xylose plus glycerol. All three of these strains exhibited faster growth than WT in this condition and the fastest growing colony was selected for further characterization as the OE-Pnt1 strain.

For fatty alcohol production, the parental strain was *R.* *toruloides* IFO 0880 with a single copy of the *Marinobacter* *aquaeolei* fatty-acyl-CoA reductase (Maqu FAR 2220, [12]) integrated at the Ku70 locus, further engineered to reduce fatty alcohol catabolism (manuscript in preparation). To create the ∆11990 OE-Pnt1 fatty alcohol production strain, the parental strain was transformed with a linearized plasmid containing the OE-Ptn1 expression cassette flanked by homologous sequences to the RTO4_11990 locus. Outside the RTO4_11990 sequence was a GFP fluorescence cassette. A non-GFP expressing transformant was confirmed for OE-Pnt1 integration at the RTO4_11990 locus with diagnostic PCR. Three independent GFP positive transformants were also selected as random integration mutants, with a presumably intact RTO4_11990 locus, though that sequence was not confirmed.

### Media and growth conditions

All chemicals used in this study were from Sigma Aldrich unless otherwise stated. Strains were cultured at 30 °C unless otherwise stated. For regular strain maintenance and transformation, cells were grown in 10 g/L yeast extract, 20 g/L peptone, 20 g/L glucose (YPD).

For growth and sugar utilization experiments (Figs. 2, 3), strains were grown on medium consisting of 40 g/L xylose (or 20 g/L xylose plus 20 g/L glucose) with 1.7 g/L yeast nitrogen base without ammonium sulfate and amino acids (BD 233520), 0.2 g/L MgCl_2_⋅6H_2_O, 0.25 g/L MgSO_4_⋅6H_2_O, 2.5 mM KCl, 10 g/L yeast extract (Y1625), and 100 µM FeSO_4_ (final pH 5.6). This medium formulation is similar to one previously reported to maximize *R.* *toruloides* xylose utilization [51]. With 40 g/L xylose, and assuming that yeast extract has a similar C/N ratio to glutathione, this medium has a C/N ratio ~ 17, which should result in relatively low lipid accumulation. Cells were precultured in YPD overnight, washed with water, inoculated at an optical density of 0.05 or 0.025 (for xylose and glucose plus xylose conditions, respectively) in 600 µL experimental medium and grown for 28 h in a microtiter plate format: 48-well M2P Labs Flower Plate MTP-48-B (2000 µL total volume) at 1500 RPM agitation, 30 °C, and 85% relative humidity in a BioLector Pro high-throughput microbioreactor (M2P Labs). The M2P Labs Flower Plate allows for small scale cultivation at high oxygen transfer rates [52]. Over the course of the experiment, 20 µL samples were collected for optical density and HPLC analysis of glucose and xylose. For Additional file 3: Fig. S1 the medium was the same as above except that sugar concentrations were 40 g/L xylose (C/N ~ 17) or 40 g/L xylose plus 40 g/L glucose (C/N ~ 30) and total culture volume was 400 µL. For Additional file 3: Fig. S2, medium was the same as that used for proteomics experiments, see below.

For fatty alcohol production experiments, we used a modified synthetic defined medium (1.7 g/L yeast nitrogen base, 0.79 g/L complete supplement mix, 5 g/L (NH_4_)_2_SO_4_, 100 mM phosphate buffer at pH 6.0, 10 µM FeSO_4_, and 0.1% tergitol). To this base media we added three different sugar combinations: 10 g/L xylose (C/N ~ 5); 100 g/L xylose (C/N ~ 45); or for the mock hydrolysate condition 75 g/L Glucose plus 40 g/L xylose (C/N ~ 50). For the hydrolysate condition, concentrated deacetylated, mechanically refined corn stover hydrolysate [29] was obtained from the National Renewable Energy Laboratory and diluted to 75 g/L glucose with 1 g/L ammonium sulfate (Sigma A4418), 100 mM phosphate buffer at pH 6.0, 10 uM FeSO4, and 0.1% tergitol. At this concentration the medium contains approximately 40 g/L xylose, 5 g/L arabinose, 2.5 g/L lactic acid, 0.5 g/L acetate, approximately 0.5–1 g/L of the lignin breakdown products benzoic acid, coumaric acid and ferulic acid, and approximately 1 mM amino acids (C/N ~ 250). This hydrolysate was specifically developed to reduce the concentrations of common microbial inhibitors furfural and hydroxymethylfurfural, but presumably many such inhibitory compounds were present at low levels. Cells were precultured in YPD overnight and inoculated at an optical density of 0.1 in 800 µl experimental medium plus 200 µL of dodecane overlay (Sigma D221104) and cultured in microtiter plates for 6 days. At the conclusion of the experiment, 100 µL dodecane with 1 mg/mL tridecanol (Sigma T57630) was added to the culture, then mixed vigorously. Then 60 µL of the organic overlay was sampled, diluted into 300 µl ethyl acetate (Sigma, 1007891000) and stored for GC-FID analysis. Culture supernatants were also tested for remaining xylose with the Neogen Megazyme xylose assay kit (cat# k-xylose).

For proteomic analysis the growth medium was 40 g/L xylose (or 40 g/L xylose plus 40 g/L glycerol), 6.7 g/L yeast nitrogen base with ammonium sulfate and without amino acids (Sigma Y0626), 180 mM KH_2_PO_4_, 20 mM K_2_HPO_4_, 176 mg/L nitrilotriacetic acid, 600 mg/L MgSO_4_⋅7H_2_O, 200 mg/L MgCl_2_⋅6H_2_O, 120 mg/L MnSO_4_⋅4H_2_O, 118 mg/L NaCl, 36 mg/L FeSO_4_⋅7H_2_O, 11.8 mg/L CoSO_4_⋅7H_2_O, 11.8 mg/L CaCl_2_⋅2H_2_O, 11.8 mg/L ZnSO_4_⋅7H_2_O, 1.2 mg/L CuSO_4_⋅5H_2_O, 1.2 mg/L AlK(SO_4_)_2_⋅12H_2_O, 11.8 mg/L H_3_BO_3_, Na_2_MoO_4_⋅2H_2_O, 2.5 mM KCl, pH 5.6 (C/N ~ 18). Four individual colonies for each strain were precultured in YPD overnight, washed with water, inoculated to varying initial optical densities, and grown for 30 or 56 h (for xylose and xylose plus glycerol conditions, respectively). Because different strains had different growth rates, to maintain cells within exponential phase and generate enough biomass for protein extraction and analysis, different starting inocula and culture times were required for each strain and condition. Culturing took place in 800 µL of growth medium in a 48-well M2P Labs microtiter Flower Plate (MTP-48-B) with a total of 4 biological and 4 technical replicates for each strain. Cultures were then harvested during exponential phase (average OD_600_ 5 ± 2) by sampling 6 OD_600_ (10 mm path length) equivalents for each biological replicate (after combining the 4 technical replicates for each biological replicate), centrifuged for 1 min at 21,000 RCF, supernatant decanted, pellet flash frozen with liquid nitrogen, and stored at − 80 °C for protein extraction and analysis.

### Sugar quantification

Sugars were quantified on a Dionex Ultimate 3000 system UHPLC (Agilent Technologies) using an Aminex HPX-87H column (Bio-Rad 1250140) and Thermo Scientific RefractoMax 520 Refractive Index Detector (RID) held at 35 °C. Prior to analysis, samples were diluted to 1:10 and filtered through a 0.45 µM polypropylene membrane microplate filter (Agilent 200983-100) by centrifugation at 3000 RCF for 3 min. Samples were run for 26 min using an isocratic 4 mM sulfuric acid mobile phase at 0.6 mL/min and 65 °C. Glucose, xylose, and glycerol standards were prepared and diluted to create a 5-point calibration curve ranging from 0.125 to 2.0 mg/mL.

### Fatty alcohol quantification

Fatty alcohols were quantified by gas chromatography with a DB-wax column (Agilent, 123-7012) on a Thermo Scientific Focus gas chromatograph (AS 3000 II) with a flame ionization detector. For each sample, the column was equilibrated at 150 °C for 3 min, followed by a ramp to 245 °C at 20 °C/min, and then was held at this temperature for 6 min. Final FOH concentrations were measured by comparing the peak areas of cetyl alcohol C16:0, palmitoleyl alcohol C16:1, 1‐heptadecanol C17:0, stearyl alcohol C18:0, and oleyl alcohol C18:1 to that of the C13:0 internal standard with calibration curves from a custom FOH standard mix in ethyl acetate.

### Statistical analysis

For all direct comparisons of growth, sugar utilization, or fatty alcohol accumulation described in the text, we use a 1-tailed student’s T-test on three independent biological replicates (calculated in Microsoft Excel). A table of these calculations is included in Additional file 2. For statistical analysis of proteomic data we used a combination of parametric (ANOVA with post-hoc Tukey test on intensity scores) and non-parametric tests (G-test on peptide abundance/absence) as previously described for proteomic intensity data with missing values (Webb-Robertson et al., 2010). P-values were then corrected for multiple hypothesis testing with the Benjamini–Hochberg procedure [53].

### Proteomic analysis

Proteomic samples were prepared based on a previously established protocol [4, 54]. Equal biomass cell pellets (6 OD units) were subjected to protein extraction and digestion. Equal total mass of peptide digests (500 ng) were then loaded onto in-house packed reversed-phase capillary columns (30 cm × 75 μm i.d.) with 1.7-μm Waters AQ BEH. The separation was carried out using a standard LC system equipped with a binary nanoUPLC pump (Thermo Dionex Ultimate 3000, Thermo Scientific) with trapping. The mobile phase A is 0.1% formic acid in water while mobile phase B is 0.1% formic acid in acetonitrile. The elution was carried out at 200 nL/min at 50 °C with the following gradient: 0–2.6 min 1% B; 2.6–12.6 min 1%-8% B; 12.6–107.6 min 8–12% B; 107.6–117.6 min 25–35% B; 117.8–122.6 min 35–75% B. The LC column was washed using 95% B and equilibrated using 1% B. MS analysis was carried out using a Q Exactive HF-X Orbitrap MS (Thermo Scientific, San Jose, CA, USA) in data dependent mode. Mass spectrometer settings were as following: full MS (AGC, 3 × 10^6^; resolution, 60,000; m/z range, 300–1800; maximum ion time, 20 ms); MS/MS (AGC, 1 × 10^5^; resolution, 45,000; m/z range, 200–2000; maximum ion time, 100 ms; minimum signal threshold, 5.0 × 10^3^; isolation width, 0.7 Da; TopN 12; dynamic exclusion time setting, 45 s; collision energy, NCE 30).

Protein identification was carried out by MS-GF + [55] and filtered by false discovery rate of ≤ 1% (Qvalue < 0.01 in MS-GF +) and less than 5-ppm mass accuracy. The peptide-level intensities were obtained by MASIC [56] software and used for further data processing. Data quality was ensured by a robust Principal Component Analysis to compute a robust Mahalanobis distance based on sample-level parameters [57]. The default for normalization is standard global median centering to account for total abundance differences between samples. A test was performed to assure that these factors are not biased [58]. For this dataset, no bias was detected and we utilized global median centering [59]. The objective of this normalization procedure is that intensities across samples should represent relative abundance of a given protein as a fraction of total protein in each sample. Protein quantification was performed with standard reference-based median averages [60, 61]. The data have been deposited to the ProteomeXchange Consortium [62] via the MASSIVE partner repository with the accession number of MSV000089938.

### Supplementary Information

**Additional file 1**: Intensities and statistical analysis from global proteomics of WT IFO 0880, OE-Pnt1, ∆Pnt1 grown on xylose and xylose plus glycerol medium.

**Additional file 2** Strains used in this study with strain IDs and part numbers for relevant plasmids for retrieval on the Agile BioFoundry public registry (https://public-registry.agilebiofoundry.org) Data used for T-tests referenced in the text.

**Additional file 3:** **Figure S1**. Growth, sugar utilization, and biomass yields of OE-Pnt1 and WT *R.* *toruloides* on media containing 40 g/L xylose or 40 g/L xylose plus 40 g/L glucose. (A-B) OD 600 (C-D) Remaining xylose in the medium. (E-F) Arabitol accumulation. (G-H) Xylitol accumulation. **Figure S2**. High-resolution time course of OE-Pnt1 growth vs WT on 40 g/L xylose. (A) Log biomass. Biolector absorbance measurements processed to subtract individual well bias and smoothed with a LOWESS curve before log transformation. (B) Specific growth rate calculated from smoothed biolector absorbance. Note that relation of biolector absorbance to true biomass by dry weight may change with culture density and cellular morphology. **Figure S3**. Xylose consumption in strains overexpressing Pnt1 from the Tef1 promoter, either integrated at the RTO4_11990 locus, or randomly integrated into the genome. (A) 10 g/L xylose YNB medium. (B) 100 g/L xylose YNB medium (C) Corn stover hydrolysate: approximately 75 g/L glucose, 40 g/L xylose. (D) Mock hydrolysate with 75 g/L glucose, 40 g/L xylose in YNB medium. P-values from a student’s T-test vs the parent strain are shown for each strain. **Figure S4**. Principal component analysis of protein intensities from global proteomics of WT IFO 0880, OE-Pnt1, ∆Pnt1 strains grown on xylose or xylose plus glycerol. Log_2_-transformed intensities were normalized across samples by z-score before PCA analysis. **Figure S5**. Metabolic model of *R.* *toruloides* carbon metabolism with relative protein abundance in the OE-Pnt1 strain and ∆Pnt1 strain on xylose plus glycerol. Reaction arrows are mean log_2_-fold change of all proteins with predicted function in the *R.* *toruloides* metabolic model. **Figure S6**. Hierarchical clustering of intensity Z-scores for 58 differentially abundant proteins in Pnt1 mutants grown on xylose. Proteins included in this analysis had at least 2-fold differential abundance in OE-Pnt1 vs ∆Pnt1 on both xylose and xylose plus glycerol. They also were required to show consistent statistically significant (P-value < 0.05) abundance differences to WT in both mutant strains in both media conditions. Statistical significance was assessed with a multiple-hypothesis-adjusted, equal-variance two-tailed T-test on protein intensities. Proteins and samples were bidirectionally clustered with Euclidean distance as the similarity metric and the Ward method for clustering. White cells are samples in which a protein was not detected.

## Data Availability

The dataset supporting the conclusions of this article is available in the ProteomeXchange Consortium repository, in http://proteomecentral.proteomexchange.org/cgi/GetDataset?ID=PXD035507 and included within the article (and its additional files). All strains named in this work are available to order through the Agile BioFoundry parts registry at https://public-registry.agilebiofoundry.org. The registry website also hosts all applicable plasmid sequences.

## References

[1] Houfani AA, Anders N, Spiess AC, Baldrian P, Benallaoua S (2020). Insights from enzymatic degradation of cellulose and hemicellulose to fermentable sugars—a review. Biomass Bioenerg.

[2] Chavan S, Yadav B, Atmakuri A, Tyagi RD, Wong JWC, Drogui P (2021). Bioconversion of organic wastes into value-added products: a review. Bioresour Technol.

[3] Li X, Chen Y, Nielsen J (2019). Harnessing xylose pathways for biofuels production. Curr Opin Biotechnol.

[4] Kim J, Coradetti ST, Kim Y-M, Gao Y, Yaegashi J, Zucker JD (2020). Multi-omics driven metabolic network reconstruction and analysis of lignocellulosic carbon utilization in *Rhodosporidium*
*toruloides*. Front Bioeng Biotechnol.

[5] Saini R, Hegde K, Osorio-Gonzalez CS, Brar SK, Vezina P (2020). Evaluating the potential of *Rhodosporidium*
*toruloides*-1588 for high lipid production using undetoxified wood hydrolysate as a carbon source. Energies.

[6] Liu Z, Radi M, Mohamed ETT, Feist AM, Dragone G, Mussatto SI (2021). Adaptive laboratory evolution of *Rhodosporidium*
*toruloides* to inhibitors derived from lignocellulosic biomass and genetic variations behind evolution. Bioresour Technol.

[7] Lopes HJS, Bonturi N, Miranda EA (2021). Induction of resistance mechanisms in *Rhodotorula*
*toruloides* for growth in sugarcane hydrolysate with high inhibitor content. Appl Microbiol Biotechnol.

[8] Geiselman GM, Kirby J, Landera A, Otoupal P, Papa G, Barcelos C (2020). Conversion of poplar biomass into high-energy density tricyclic sesquiterpene jet fuel blendstocks. Microb Cell Fact.

[9] Zhao Y, Song B, Li J, Zhang J (2021). *Rhodotorula*
*toruloides*: an ideal microbial cell factory to produce oleochemicals, carotenoids, and other products. World J Microbiol Biotechnol.

[10] Wehrs M, Gladden JM, Liu Y, Platz L, Prahl J-P, Moon J (2019). Sustainable bioproduction of the blue pigment indigoidine: expanding the range of heterologous products in *R.*
*toruloides* to include non-ribosomal peptides. Green Chem.

[11] Yaegashi J, Kirby J, Ito M, Sun J, Dutta T, Mirsiaghi M (2017). *Rhodosporidium*
*toruloides*: a new platform organism for conversion of lignocellulose into terpene biofuels and bioproducts. Biotechnol Biofuels.

[12] Liu D, Geiselman GM, Coradetti S, Cheng Y-F, Kirby J, Prahl J-P (2020). Exploiting nonionic surfactants to enhance fatty alcohol production in *Rhodosporidium*
*toruloides*. Biotechnol Bioeng.

[13] Wiebe MG, Koivuranta K, Penttilä M, Ruohonen L (2012). Lipid production in batch and fed-batch cultures of *Rhodosporidium*
*toruloides* from 5 and 6 carbon carbohydrates. BMC Biotechnol.

[14] Monteiro de Oliveira P, Aborneva D, Bonturi N, Lahtvee P-J (2021). Screening and growth characterization of non-conventional yeasts in a hemicellulosic hydrolysate. Front Bioeng Biotechnol..

[15] Farwick A, Bruder S, Schadeweg V, Oreb M, Boles E (2014). Engineering of yeast hexose transporters to transport d-xylose without inhibition by d-glucose. Proc Natl Acad Sci USA.

[16] Broach JR (2012). Nutritional control of growth and development in yeast. Genetics.

[17] MacPherson S, Larochelle M, Turcotte B (2006). A fungal family of transcriptional regulators: the zinc cluster proteins. Microbiol Mol Biol Rev.

[18] Wu VW, Thieme N, Huberman LB, Dietschmann A, Kowbel DJ, Lee J (2020). The regulatory and transcriptional landscape associated with carbon utilization in a filamentous fungus. Proc Natl Acad Sci USA.

[19] Vik A, Rine J (2001). Upc2p and Ecm22p, dual regulators of sterol biosynthesis in *Saccharomyces*
*cerevisiae*. Mol Cell Biol.

[20] Jansuriyakul S, Somboon P, Rodboon N, Kurylenko O, Sibirny A, Soontorngun N (2016). The zinc cluster transcriptional regulator Asg1 transcriptionally coordinates oleate utilization and lipid accumulation in *Saccharomyces*
*cerevisiae*. Appl Microbiol Biotechnol.

[21] Holmberg S, Schjerling P (1996). Cha4p of *Saccharomyces*
*cerevisiae* activates transcription via serine/threonine response elements. Genetics.

[22] Rzechonek DA, Neuvéglise C, Devillers H, Rymowicz W, Mirończuk AM (2017). EUF1—a newly identified gene involved in erythritol utilization in *Yarrowia*
*lipolytica*. Sci Rep.

[23] Mirończuk AM, Biegalska A, Zugaj K, Rzechonek DA, Dobrowolski A (2018). A role of a newly identified isomerase from *Yarrowia*
*lipolytica* in erythritol catabolism. Front Microbiol.

[24] Li H, Alper HS (2016). Enabling xylose utilization in *Yarrowia*
*lipolytica* for lipid production. Biotechnol J.

[25] Ozcan S, Dover J, Rosenwald AG, Wölfl S, Johnston M (1996). Two glucose transporters in *Saccharomyces*
*cerevisiae* are glucose sensors that generate a signal for induction of gene expression. Proc Natl Acad Sci USA.

[26] Wang B, Li J, Gao J, Cai P, Han X, Tian C (2017). Identification and characterization of the glucose dual-affinity transport system in Neurospora crassa: pleiotropic roles in nutrient transport, signaling, and carbon catabolite repression. Biotechnol Biofuels.

[27] Wang Y, Lin X, Zhang S, Sun W, Ma S, Zhao ZK (2016). Cloning and evaluation of different constitutive promoters in the oleaginous yeast *Rhodosporidium*
*toruloides*. Yeast.

[28] Salusjärvi L, Kankainen M, Soliymani R, Pitkänen J-P, Penttilä M, Ruohonen L (2008). Regulation of xylose metabolism in recombinant *Saccharomyces*
*cerevisiae*. Microb Cell Fact.

[29] Chen X, Kuhn E, Jennings EW, Nelson R, Tao L, Zhang M (2016). DMR (deacetylation and mechanical refining) processing of corn stover achieves high monomeric sugar concentrations (230 g L^−1^) during enzymatic hydrolysis and high ethanol concentrations (>10% v/v) during fermentation without hydrolysate purification or concentration. Energy Environ Sci.

[30] Patel A, Pruthi V, Singh RP, Pruthi PA (2015). Synergistic effect of fermentable and non-fermentable carbon sources enhances TAG accumulation in oleaginous yeast *Rhodosporidium*
*kratochvilovae* HIMPA1. Bioresour Technol.

[31] King ZA, Dräger A, Ebrahim A, Sonnenschein N, Lewis NE, Palsson BO (2015). Escher: a web application for building, sharing, and embedding data-rich visualizations of biological pathways. PLoS Comput Biol.

[32] Eaton RW (1997). p-Cymene catabolic pathway in Pseudomonas putida F1: cloning and characterization of DNA encoding conversion of p-cymene to p-cumate. J Bacteriol.

[33] Becker-Kettern J, Paczia N, Conrotte J-F, Kay DP, Guignard C, Jung PP (2016). *Saccharomyces*
*cerevisiae* Forms D-2-hydroxyglutarate and couples its degradation to d-lactate formation via a cytosolic transhydrogenase. J Biol Chem.

[34] Olgeiser L, Haag C, Boerner S, Ule J, Busch A, Koepke J, et al. The key protein of endosomal mRNP transport Rrm4 binds translational landmark sites of cargo mRNAs. EMBO Rep. 2019;20(1). 10.15252/embr.201846588 PMC6322384 30552148

[35] Roca C, Nielsen J, Olsson L (2003). Metabolic engineering of ammonium assimilation in xylose-fermenting *Saccharomyces*
*cerevisiae* improves ethanol production. Appl Environ Microbiol.

[36] Guo W, Sheng J, Zhao H, Feng X (2016). Metabolic engineering of *Saccharomyces*
*cerevisiae* to produce 1-hexadecanol from xylose. Microb Cell Fact.

[37] Wang W, Wei H, Knoshaug E, Van Wychen S, Xu Q, Himmel ME (2016). Fatty alcohol production in *Lipomyces*
*starkeyi* and *Yarrowia*
*lipolytica*. Biotechnol Biofuels.

[38] Zhang S, Ito M, Skerker JM, Arkin AP, Rao CV (2016). Metabolic engineering of the oleaginous yeast *Rhodosporidium*
*toruloides* IFO0880 for lipid overproduction during high-density fermentation. Appl Microbiol Biotechnol.

[39] Cordova LT, Butler J, Alper HS (2020). Direct production of fatty alcohols from glucose using engineered strains of *Yarrowia*
*lipolytica*. Metab Eng Commun.

[40] Fillet S, Gibert J, Suárez B, Lara A, Ronchel C, Adrio JL (2015). Fatty alcohols production by oleaginous yeast. J Ind Microbiol Biotechnol.

[41] d’Espaux L, Ghosh A, Runguphan W, Wehrs M, Xu F, Konzock O (2017). Engineering high-level production of fatty alcohols by *Saccharomyces*
*cerevisiae* from lignocellulosic feedstocks. Metab Eng.

[42] UniProt Consortium (2021). UniProt: the universal protein knowledgebase in 2021. Nucleic Acids Res.

[43] Katoh K, Misawa K, Kuma K, Miyata T (2002). MAFFT: a novel method for rapid multiple sequence alignment based on fast Fourier transform. Nucleic Acids Res.

[44] Price MN, Dehal PS, Arkin AP (2009). FastTree: computing large minimum evolution trees with profiles instead of a distance matrix. Mol Biol Evol.

[45] Letunic I, Bork P (2021). Interactive Tree Of Life (iTOL) v5: an online tool for phylogenetic tree display and annotation. Nucleic Acids Res.

[46] Grigoriev IV, Nikitin R, Haridas S, Kuo A, Ohm R, Otillar R (2014). MycoCosm portal: gearing up for 1000 fungal genomes. Nucleic Acids Res.

[47] Alexander WG, Doering DT, Hittinger CT (2014). High-efficiency genome editing and allele replacement in prototrophic and wild strains of Saccharomyces. Genetics.

[48] Koh CMJ, Liu Y, Du Moehninsi, M, Ji L (2014). Molecular characterization of KU70 and KU80 homologues and exploitation of a KU70-deficient mutant for improving gene deletion frequency in *Rhodosporidium*
*toruloides*. BMC Microbiol.

[49] Otoupal PB, Ito M, Arkin AP, Magnuson JK, Gladden JM, Skerker JM. Multiplexed CRISPR-Cas9-based genome editing of *Rhodosporidium**toruloides*. mSphere. 2019;4(2). 10.1128/mSphere.00099-19 PMC6429044 30894433

[50] Coradetti ST, Pinel D, Geiselman GM, Ito M, Mondo SJ, Reilly MC, et al. Functional genomics of lipid metabolism in the oleaginous yeast *Rhodosporidium**toruloides*. eLife. 2018;7. 10.7554/eLife.32110 PMC5922974 29521624

[51] Jagtap SS, Rao CV (2018). Production of d-arabitol from d-xylose by the oleaginous yeast *Rhodosporidium*
*toruloides* IFO0880. Appl Microbiol Biotechnol.

[52] Funke M, Diederichs S, Kensy F, Müller C, Büchs J (2009). The baffled microtiter plate: increased oxygen transfer and improved online monitoring in small scale fermentations. Biotechnol Bioeng.

[53] Benjamini Y, Hochberg Y (1995). Controlling the false discovery rate: a practical and powerful approach to multiple testing. J Roy Stat Soc: Ser B (Methodol).

[54] Nakayasu ES, Nicora CD, Sims AC, Burnum-Johnson KE, Kim Y-M, Kyle JE, et al. MPLEx: a robust and universal protocol for single-sample integrative proteomic, metabolomic, and lipidomic analyses. mSystems. 2016;1(3). 10.1128/mSystems.00043-16 PMC5069757 27822525

[55] Kim S, Pevzner PA. MS-GF+ makes progress towards a universal database search tool for proteomics Nature Commun. 2014;5(1) 10.1038/ncomms6277 PMC5036525 25358478

[56] Monroe MW, Shaw JL, Daly DS, Adkins JN, Smith RD (2008). MASIC: A software program for fast quantitation and flexible visualization of chromatographic profiles from detected LC–MS(/MS) features. Comput Biol Chem.

[57] Matzke MM, Waters KM, Metz TO, Jacobs JM, Sims AC, Baric RS (2011). Improved quality control processing of peptide-centric LC–MS proteomics data. Bioinformatics.

[58] Webb-Robertson B-JM, McCue LA, Waters KM, Matzke MM, Jacobs JM, Metz TO (2010). Combined statistical analyses of peptide intensities and peptide occurrences improves identification of significant peptides from MS-based proteomics data. J Proteome Res.

[59] Callister SJ, Barry RC, Adkins JN, Johnson ET, Qian WJ, Webb-Robertson BJ, Smith RD, Lipton MS (2006). Normalization approaches for removing systematic biases associated with mass spectrometry and label-free proteomics. J Proteome Res.

[60] Polpitiya AD, Qian WJ, Jaitly N, Petyuk VA, Adkins JN, Camp DG, Anderson GA, Smith RD (2008). DAnTE: a statistical tool for quantitative analysis of -omics data. Bioinformatics.

[61] Matzke MM, Brown JN, Gritsenko MA, Metz TO, Pounds JG, Rodland KD, Shukla AK, Smith RD, Waters KM, McDermott JE, Webb-Robertson BJ (2013). A comparative analysis of computational approaches to relative protein quantification using peptide peak intensities in label-free LC-MS proteomics experiments. Proteomics.

[62] Vizcaíno JA, Deutsch EW, Wang R, Csordas A, Reisinger F, Ríos D, Dianes JA, Sun Z, Farrah T, Bandeira N, Binz PA, Xenarios I, Eisenacher M, Mayer G, Gatto L, Campos A, Chalkley RJ, Kraus HJ, Albar JP, Martinez-Bartolomé S, Apweiler R, Omenn GS, Martens L, Jones AR, Hermjakob H (2013). ProteomeXchange provides globally coordinated proteomics data submission and dissemination. Nature Biotechnol.

