# Supplementary material for: Engineering transcriptional regulation of pentose metabolism in Rhodosporidium toruloides for improved conversion of xylose to bioproducts
Source: Microb Cell Fact. 2023 Aug 3;22:144. doi: 10.1186/s12934-023-02148-5 (PMC10398944; doi:10.1186/s12934-023-02148-5)
Supplement: Supplementary file 3 — Additional file 3: Figure S1. Growth, sugar utilization, and biomass yields of OE-Pnt1 and WT R. toruloides on media containing 40 g/L xylose or 40 g/L xylose plus 40 g/L glucose. (A-B) OD 600 (C-D) Remaining xylose in the medium. (E-F) Arabitol accumulation. (G-H) Xylitol accumulation. Figure S2. High-resolution time course of OE-Pnt1 growth vs WT on 40 g/L xylose. (A) Log biomass. Biolector absorbance measurements processed to subtract individual well bias and smoothed with a LOWESS curve before log transformation. (B) Specific growth rate calculated from smoothed biolector absorbance. Note that relation of biolector absorbance to true biomass by dry weight may change with culture density and cellular morphology. Figure S3. Xylose consumption in strains overexpressing Pnt1 from the Tef1 promoter, either integrated at the RTO4_11990 locus, or randomly integrated into the genome. (A) 10 g/L xylose YNB medium. (B) 100 g/L xylose YNB medium (C) Corn stover hydrolysate: approximately 75 g/L glucose, 40 g/L xylose. (D) Mock hydrolysate with 75 g/L glucose, 40 g/L xylose in YNB medium. P-values from a student’s T-test vs the parent strain are shown for each strain. Figure S4. Principal component analysis of protein intensities from global proteomics of WT IFO 0880, OE-Pnt1, ∆Pnt1 strains grown on xylose or xylose plus glycerol. Log2-transformed intensities were normalized across samples by z-score before PCA analysis. Figure S5. Metabolic model of R. toruloides carbon metabolism with relative protein abundance in the OE-Pnt1 strain and ∆Pnt1 strain on xylose plus glycerol. Reaction arrows are mean log2-fold change of all proteins with predicted function in the R. toruloides metabolic model. Figure S6. Hierarchical clustering of intensity Z-scores for 58 differentially abundant proteins in Pnt1 mutants grown on xylose. Proteins included in this analysis had at least 2-fold differential abundance in OE-Pnt1 vs ∆Pnt1 on both xylose and xylose plus glycerol. They also were r [file 12934_2023_2148_MOESM3_ESM.docx]

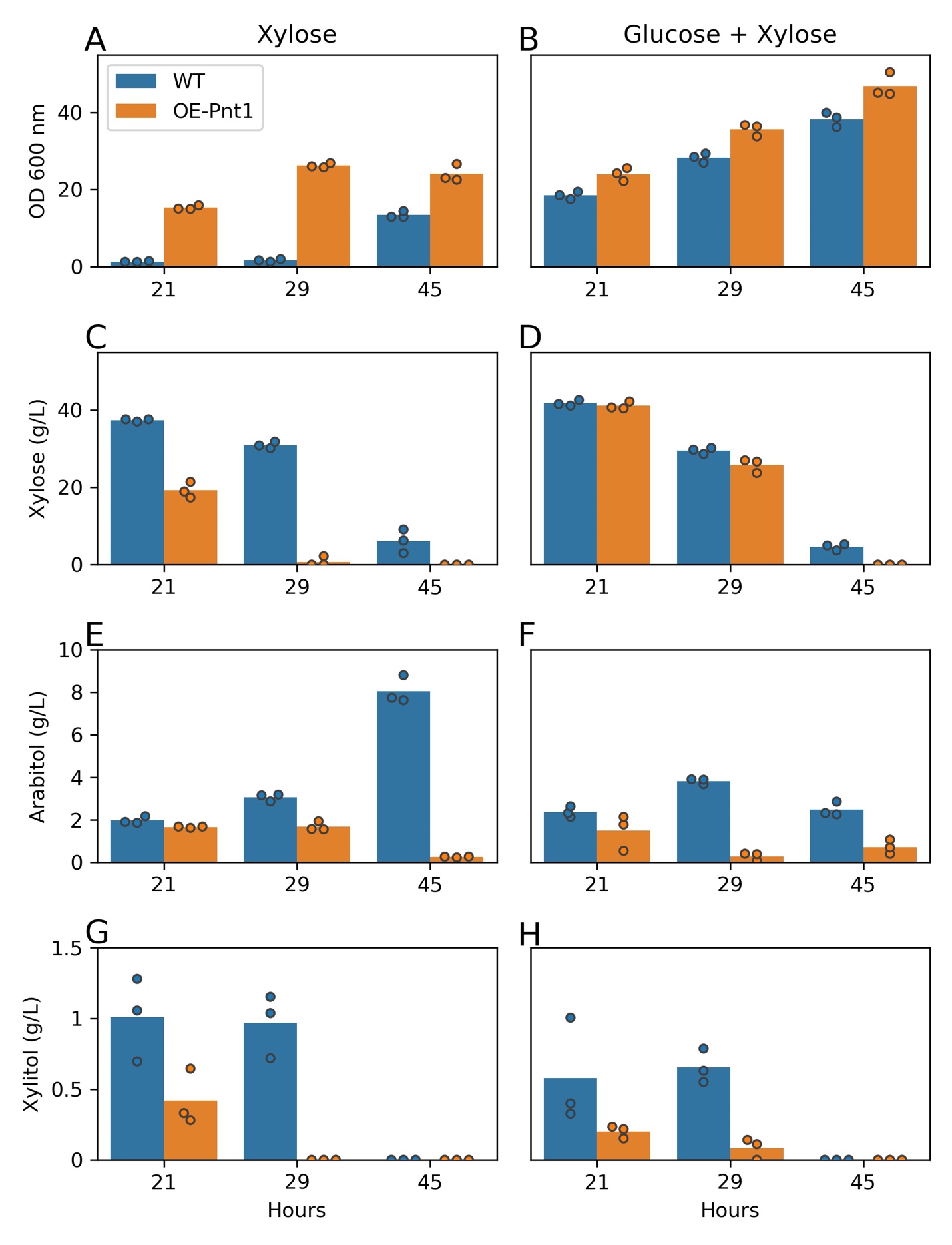

Figure S1. Growth, sugar utilization, and biomass yields of OE-Pnt1 and WT *R. toruloides* on media containing 40 g/L xylose or 40 g/L xylose plus 40 g/L glucose. (A-B) OD 600 (C-D) Remaining xylose in the medium. (E-F) Arabitol accumulation. (G-H) Xylitol accumulation.


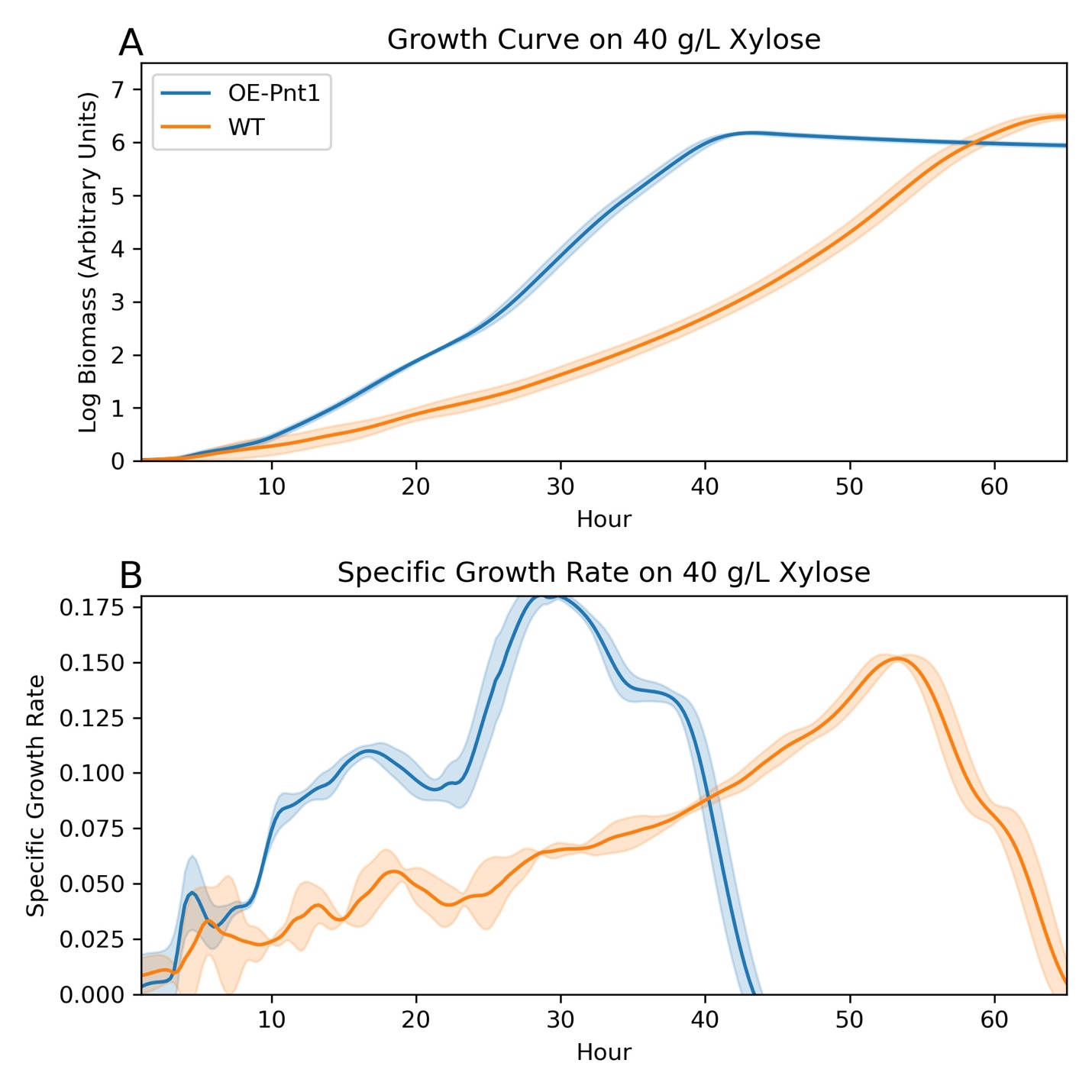

Figure S2. High-resolution time course of OE-Pnt1 growth vs WT on 40 g/L xylose. (A) Log biomass. Biolector absorbance measurements processed to subtract individual well bias and smoothed with a LOWESS curve before log transformation. (B) Specific growth rate calculated from smoothed biolector absorbance. Note that relation of biolector absorbance to true biomass by dry weight may change with culture density and cellular morphology.


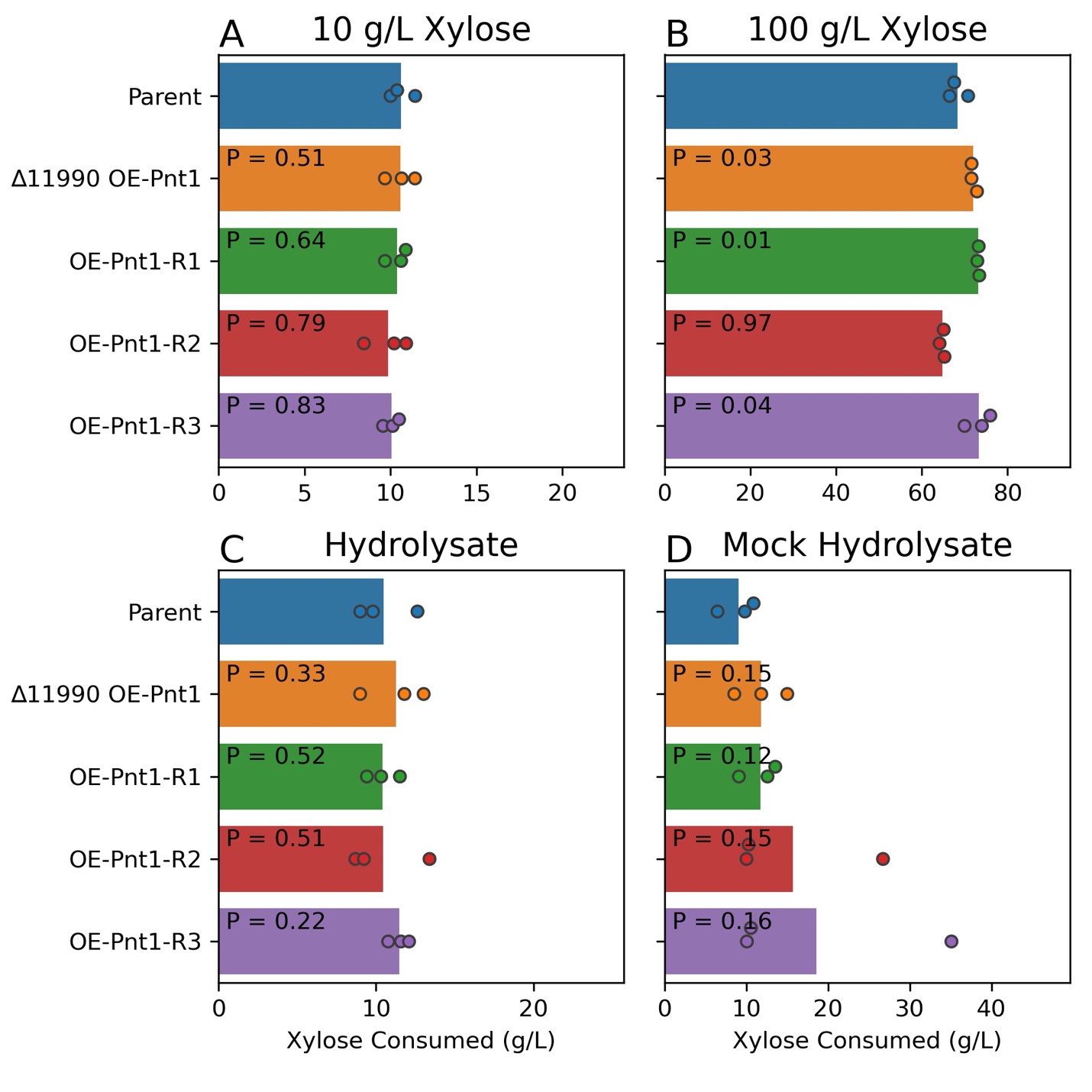

Figure S3. Xylose consumption in strains overexpressing Pnt1 from the Tef1 promoter, either integrated at the RTO4_11990 locus, or randomly integrated into the genome. (A) 10 g/L xylose YNB medium. (B) 100 g/L xylose YNB medium (C) Corn stover hydrolysate: approximately 75 g/L glucose, 40 g/L xylose. (D) Mock hydrolysate with 75 g/L glucose, 40 g/L xylose in YNB medium. P-values from a student’s T-test vs the parent strain are shown for each strain.


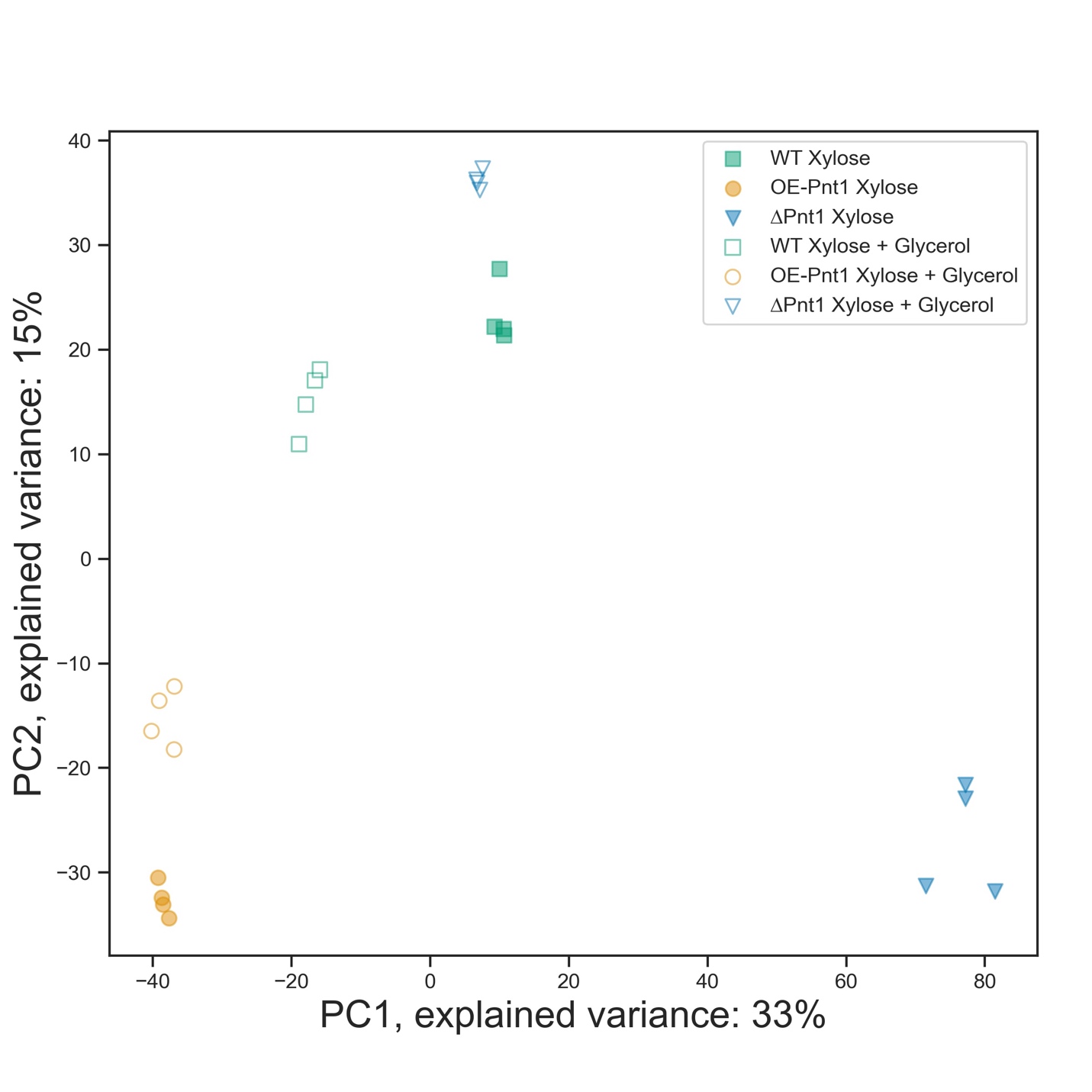

Figure S4. Principal component analysis of protein intensities from global proteomics of WT IFO 0880, OE-Pnt1, ∆Pnt1 strains grown on xylose or xylose plus glycerol. Log_2_-transformed intensities were normalized across samples by z-score before PCA analysis.


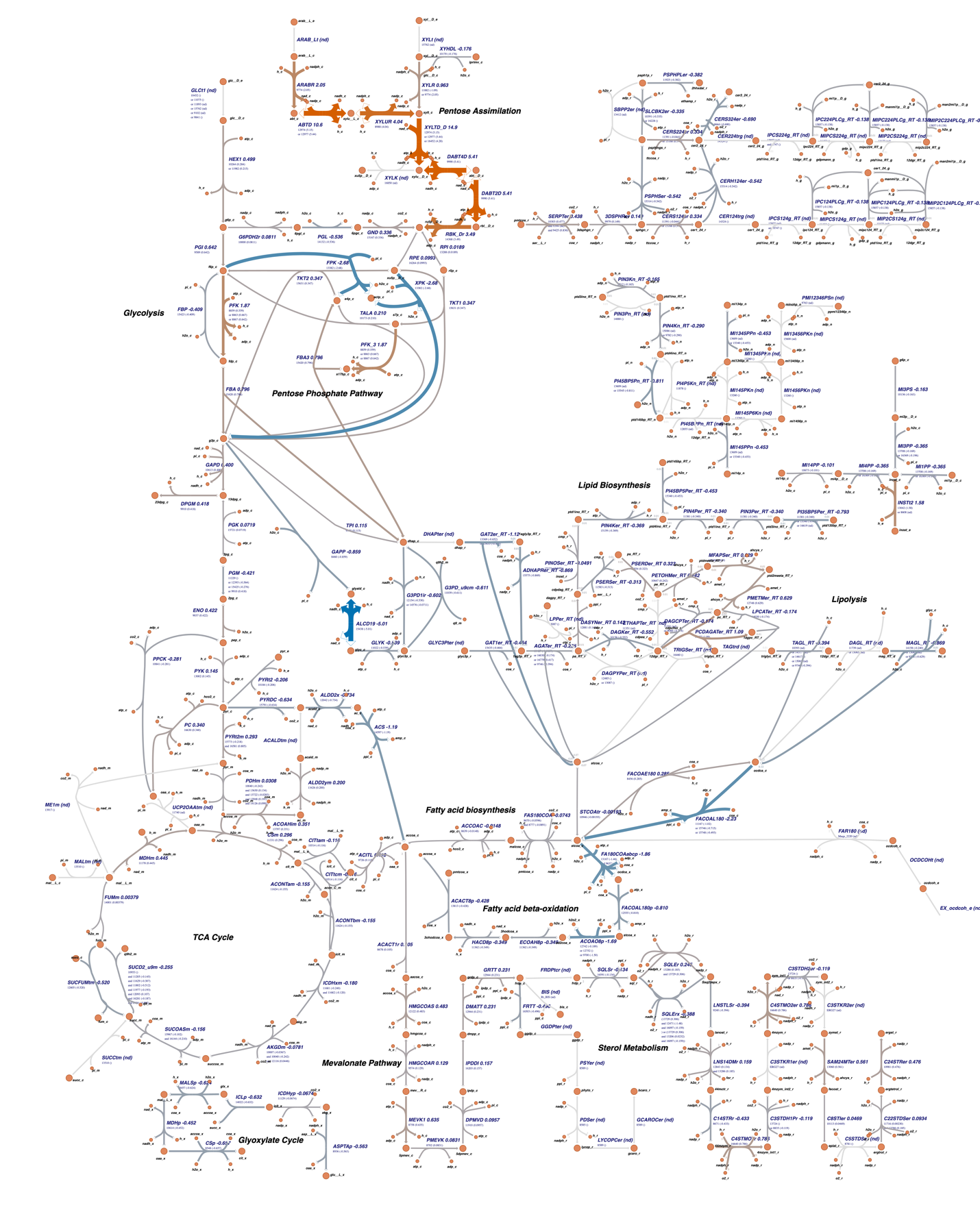

Figure S5. Metabolic model of *R. toruloides* carbon metabolism with relative protein abundance in the OE-Pnt1 strain and ∆Pnt1 strain on xylose plus glycerol. Reaction arrows are mean log_2_-fold change of all proteins with predicted function in the *R. toruloides* metabolic model. Figure made with Escher [(31)](https://sciwheel.com/work/citation?ids=832300&pre=&suf=&sa=0).


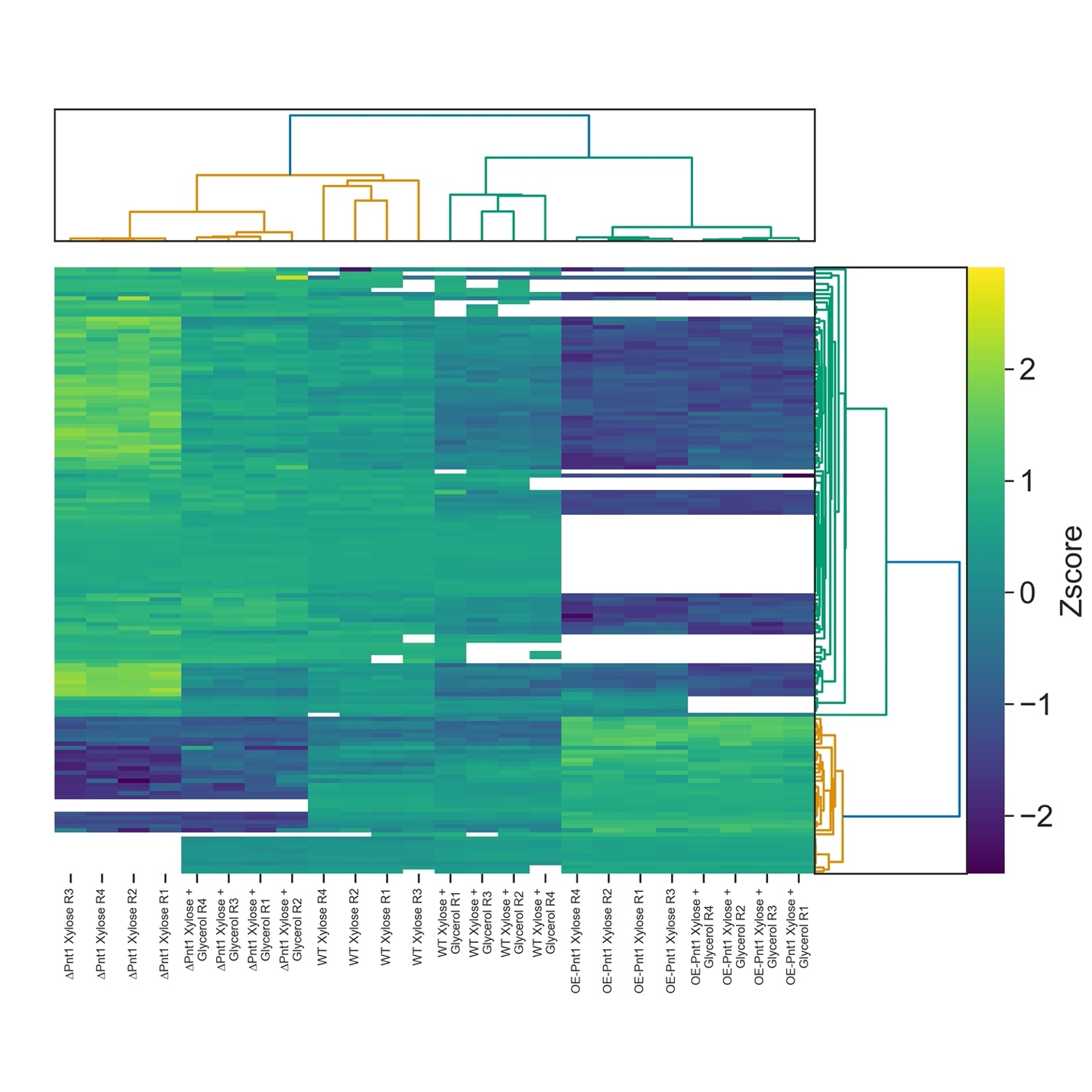

Figure Sa6: Hierarchical clustering of intensity Z-scores for 58 differentially abundant proteins in Pnt1 mutants grown on xylose. Proteins included in this analysis had at least 2-fold differential abundance in OE-Pnt1 vs ∆Pnt1 on both xylose and xylose plus glycerol. They also were required to show consistent statistically significant (P-value < 0.05) abundance differences to WT in both mutant strains in both media conditions. Statistical significance was assessed with a multiple-hypothesis-adjusted, equal-variance two-tailed T-test on protein intensities. Proteins and samples were bidirectionally clustered with Euclidean distance as the similarity metric [(32)](https://sciwheel.com/work/citation?ids=13355167&pre=&suf=&sa=0) and the Ward method [(33)](https://sciwheel.com/work/citation?ids=1173304&pre=&suf=&sa=0) for clustering. White cells are samples in which a protein was not detected.
